# Molecular selection of soybean towards adaptation to Central European agroclimatic conditions

**DOI:** 10.1007/s13353-024-00889-6

**Published:** 2024-07-02

**Authors:** Sandra Rychel-Bielska, Michał Książkiewicz, Danuta Kurasiak-Popowska, Agnieszka Tomkowiak, Wojciech Bielski, Dorota Weigt, Janetta Niemann, Anna Surma, Bartosz Kozak, Jerzy Nawracała

**Affiliations:** 1 Department of Genetics, Plant Breeding and Seed Production, Wroclaw University of Environmental and Life Sciences, 50-363 Wrocław, Poland; 2 Department of Gene Structure and Function, Institute of Plant Genetics, Polish Academy of Sciences, 60-479 Poznań, Poland; 3 Department of Genetics and Plant Breeding, Faculty of Agronomy, Horticulture and Bioengineering, Poznań University of Life Sciences, 60-632 Poznań, Poland

**Keywords:** Early maturity, Growth determination, Yield, First pod height, Marker-assisted selection

## Abstract

Europe is highly dependent on soybean meal imports and anticipates an increase of domestic plant protein production. Ongoing climate change resulted in northward shift of plant hardiness zones, enabling spring-sowing of freezing-sensitive crops, including soybean. However, it requires efficient reselection of germplasm adapted to relatively short growing season and long-day photoperiod. In the present study, a PCR array has been implemented, targeting early maturity (*E1*–*E4*, *E7*, *E9*, and *E10*), pod shattering (*qPHD1*), and growth determination (*Dt1*) genes. This array was optimized for routine screening of soybean diversity panel (204 accessions), subjected to the 2018–2020 survey of phenology, morphology, and yield-related traits in a potential cultivation region in Poland. High broad-sense heritability (0.84–0.88) was observed for plant height, thousand grain weight, maturity date, and the first pod height. Significant positive correlations were identified between the number of seeds and pods per plant, between these two traits and seed yield per plant as well as between flowering, maturity, plant height, and first pod height. PCR array genotyping revealed high genetic diversity, yielding 98 allelic combinations. The most remarkable correlations were identified between flowering and *E7* or *E1*, between maturity and *E4* or *E7* and between plant height and *Dt1* or *E4*. The study demonstrated high applicability of this PCR array for molecular selection of soybean towards adaptation to Central Europe, designating recessive *qPHD1* and dominant *Dt1*, *E3*, and *E4* alleles as major targets to align soybean growth season requirements with the length of the frost-free period, improve plant performance, and increase yield.

**Supplementary Information:**

The online version contains supplementary material available at 10.1007/s13353-024-00889-6.

## Introduction

Soybean (*Glycine max* [L.] Merrill.) is one of the most important crops worldwide, sown in more than 100 countries. It is the main domesticated legume species with cultivated area of roughly 127 million hectares and more than 350 million tons of production (FAOSTAT, 2022). Soybean is also primary source of valuable plant protein and the second source of oil supply. Although the majority of its yield is currently exploited for animal feed, soybean is considered as a key plant that may help to address the protein and caloric needs of growing global human population (Messina 2022). Poland, like other European countries, is highly dependent on soybean meal imports and awaits diversification of plant protein sources, including increase of domestic production (de Visser et al. 2014). The main reason of restricted soybean acreage in Poland despite early introduction attempts was the location of the country between 49°00′ and 54°50′ N of latitude, north of the main soybean cultivation regions (Scott and Aldrich 1983). Moreover, Poland used to have a relatively short thermal growing season (Kępinska-Kasprzak and Mager 2015) hardly fulfilling soybean climatic requirements. Nevertheless, ongoing climate change, reflected by the raise of air temperature by 0.3 °C per decade in Poland (IMGW-PIB 2022; Tomczyk and Szyga-Pluta 2019), resulted in shift of agro-climate zones about 500 km to the north during the recent 50 years (Ceglar et al. 2019), enabling spring-sown cultivation of crops natively originating from warmer regions, such as soybean, likely domesticated in Central China region under temperate and subtropical monsoon climates (Sedivy et al. 2017; Wang et al. 2023). However, further spread of soybean cultivation across Central Europe would require preselection of germplasm adapted to long-day photoperiod and local agroclimate.

Long history of soybean cultivation in different agro-ecological niches has led to a huge variability in its phenological forms, growth habit, and pod dehiscence (Hymowitz 1970). More than a dozen of early flowering/maturity loci have been identified in soybean hitherto, such as *E1* and *E2* (Bernard 1971), *E3* (Buzzell 1971), *E4* (Buzzell and Voldeng 1980), *E5* (McBlain and Bernard 1987), *E6* (Bonato and Vello 1999), *E7* (Cober and Voldeng 2001), *E8* (Cober and Morrison 2010), *E9* (Kong et al. 2014), *E10* (Samanfar et al. 2017), *E11* (Wang et al. 2019), *qDTF-J1* (Yamaguchi et al. 2014), and *J* (Ray et al. 1995). Usually, recessive alleles confer early flowering/maturity, except those of *E9* and *E11* genes. For majority of early maturity loci, particular genes have already been assigned, including phytochrome A and B photoreceptors (loci *E3* and *E4*), *FLOWERING LOCUS T* (*FT*) homologs (loci *E9*, *E10*, and *qDTF-J1*), transcription factor acting upstream of *FT* genes (locus *E1*), pleiotropic regulatory gene *GIGANETA* (locus *E2*), and an *EARLY FLOWERING 3* (*ELF3*) gene from photoperiodic pathway (locus *J*) (Liu et al. 2008; Lu et al. 2017; Samanfar et al. 2017; Takeshima et al. 2016; Tsubokura et al. 2013a; Watanabe et al. 2009, 2011; Xia et al. 2012; Zhao et al. 2016). Recessive alleles of early maturity genes usually provide earliness due to lack of function of major flowering repressors from photoperiodic pathway. Deciphered early maturity mutations include large insertions/deletions (encompassing also repetitive elements causing epigenetic silencing) as well as single nucleotide polymorphisms (SNPs), such as deletions or substitutions resulting in frameshifts, premature stop codons, or alterations of amino acid sequence in key functional domains (Liu et al. 2008; Lu et al. 2017; Samanfar et al. 2017; Takeshima et al. 2016; Tsubokura et al. 2013a; Watanabe et al. 2009, 2011; Xia et al. 2012). The only exemption is *E9* gene, encoding an ortholog of Arabidopsis *FLOWERING LOCUS T*, named in soybean as *FT2a*. It is flowering activator and its recessive allele delays flowering because of lower transcript abundance due to the insertion of *Ty1/copia-like* retrotransposon, *SORE-1* (Zhao et al. 2016).

Moreover, two major genes controlling growth habit were described in soybean, *Dt1* for determinate growth (recessive allele) (Liu et al. 2010; Tian et al. 2010) and *Dt2* for semi-determinate (dominant allele) (Liu et al. 2016; Ping et al. 2014); however, candidate functional mutations were provided only for *Dt1*, in the form of several independent non-synonymous substitutions in the *TERMINAL FLOWER1* (*TFL1*) gene. Soybean, as many other legume species, evolved explosive pod dehiscence at maturity as a method for effective seed dispersal (Parker et al. 2021). While advantageous in natural population, this trait is deleterious in crop cultivation and as such was targeted during early domestication of the species. Reduced pod shattering in cultivated soybean is related with the presence of domestication-related gene *SHAT1-5* that activates secondary cell-wall biosynthesis and promotes the thickening of the dehiscence site in soybean pods (Dong et al. 2014). Nevertheless, domesticated *SHAT1-5* allele does not provide full non-shattering in dry conditions, contrary to the domesticated non-functional (premature stop codon) allele of the *qPDH1* gene that encodes a dirigent-like protein responsible for increasing torsion of pod walls under low humidity (Funatsuki et al. 2014). The *qPDH1* gene was supplemented with KSS-SNP5 marker, recently validated for molecular-assisted selection of pod shattering–resistant germplasm (Kim et al. 2020; Lee et al. 2017).

The lack of information about germplasm suitable for adaptation in Central Europe climate, characterized by northern latitudes, is one of the main factors hampering soybean breeding in this region. Moreover, there is an issue of narrow gene pool in candidate germplasm imported from other regions, which results from several genetic bottlenecks that occurred during soybean domestication (Gizlice et al. 1994; Hyten et al. 2006; Zhuang et al. 2022). To avoid further loss of genetic diversity, soybean breeding in Europe should be based on a wide range of germplasm resources, carrying different combinations of early maturity loci, supplemented with alleles for non-shattering pods and indeterminate growth type. To address this issue, marker array for early maturity (*E1*, *E2*, *E3*, *E4*, *E7*, *E9* and *E10*), growth determination (*Dt1*), and pod shattering (*qPDH1*) genes was optimized for routine PCR-based agarose gel screening and exploited for genotyping of soybean diversity paned gathered at the Department of Genetics and Plant Breeding of Poznan University of Life Sciences in Poland. Information on allelic composition of genes conferring early maturity, growth determination, and pod shattering was confronted with the results of 3-year field observation series of phenology, morphology, and yield-related traits in a candidate soybean cultivation region in Poland to designate high-priority alleles for molecular selection.

## Materials and methods

### Plant material

Soybean diversity panel was constructed to represent wide profile of maturity groups, ranging from 000 to III. Moreover, accessions with known allelic composition of early maturity genes were included, targeting different combinations of recessive and dominant alleles at major loci (*E1*-*E4*). The panel was composed of germplasm retrieved from the following germplasm resources: Soybean Germplasm Collection located at Soybean/maize Germplasm, Pathology, and Genetics Research unit of the United States Department of Agriculture (USDA) in Urbana, IL, USA (43 accessions); Plant Gene Resources of Canada in Saskatchewan, Canada (51 accessions); Japanese Soybean Core Collection maintained at the National Institute of Agrobiological Sciences (NIAS, until 2016) and The Research Center of Genetic Resources of the National Agriculture and Food Research Organization (NARO, since 2016) in Tsukuba, Ibaraki, Japan (37 accessions); and Leguminous Crops Genetic Resources Department, N. I. Vavilov Research Institut of Plant Industry, St. Petersburg, Russia (1 accession). The other genotypes were selected from accessions gathered in early years at the Department of Genetics and Plant Breeding (DG&PB) of the Poznań University of Life Sciences, Poznań, Poland: 48 cultivars developed in European countries, 16 breeding lines developed in DG&PB, and 7 Polish varieties—4 obtained in DG&PB, 1 in DANKO Plant Breeding Ltd., 1 in Plant Breeding Strzelce Ltd, and 1 in Plant Breeding and Acclimatization Institute—National Research Institute. List of soybean accessions used in the study is provided in the Supplementary Table S1.

### PCR-based markers tagging early maturity, growth determination, and pod shattering genes

The set of molecular markers tagging early maturity (*E1*, *E2*, *E3*, *E4*, *E7*, *E9*, and *E10*), growth determination (*Dt1* and *Dt2*), and pod shattering (*qPHD1*) genes was established (Kim et al. 2020; Kong et al. 2014; Lee et al. 2017; Liu et al. 2008, 2010; Molnar et al. 2003; Ping et al. 2014; Samanfar et al. 2017; Tian et al. 2010; Tsubokura et al. 2013a, 2013b; Watanabe et al. 2009, 2011; Xia et al. 2012; Xu et al. 2013; Zhao et al. 2016). Besides using published primers, we designed new markers for two *Dt1* alleles that had not been supplied with PCR-based genotyping methodology (Liu et al. 2010; Tian et al. 2010) and one new marker for the pod shattering (*qPHD1*) gene using information on sequence polymorphism at KSS-SNP5 locus (Kim et al. 2020; Lee et al. 2017). The primers flanking these loci were designed using Primer3Plus (Untergasser et al. 2012, 2007). Depending on the availability of restriction enzymes, SNPs were resolved using the cleaved amplified polymorphic sequence (CAPS) (Konieczny and Ausubel 1993) or derived CAPS (dCAPS) (Neff et al. 1998) methods. Restriction sites and dCAPS primers were identified using dCAPS Finder 2.0 and SNP2dCAPS (Neff et al. 2002; Thiel et al. 2004). List of primers and targeted regions for growth determination, early maturity, and pod shattering genes is provided in Table 1. PCR conditions were as follows: initial denaturation (94 °C, 3 min), 35 cycles composed of three 30-s steps: denaturation (94 °C), annealing (Table 2), and elongation (72 °C), and final elongation (72 °C, 5 min).

**Table 1:** Primers and targeted regions for amplification of PCR markers for growth determination, early maturity, and pod shattering genes

Gene	No	Reference	Primer sequence	Gene
*Dt1*	1	Liu et al. (2010)	F:CCATGCTTAATCGGCATCACT R:GGTGGTGGCATAGTTTAATT	*Glyma.19g194300* promoter
	2, 3, 36	Liu et al. (2010)	F:GAGTTACAACAAGAAGCAAGTT R:GCACCGAAAAAGGGGGACATTT	*Glyma.19g194300* exon/intron 1
	4	Liu et al. (2010)	F:GGCTGCTGTCTACTTCAATGTCTAG, R:GCCACATGTGAAGATCAACTTCCA	*Glyma.19g194300* exon 4
	5, 6	Liu et al. (2010)	F:CACACCCACCCCACATATAT, R:GGCAAAACCAGCAGCTACTT	*Glyma.19g194300* exon 4
*Dt2*	7	Ping et al. (2014)	F:GGTGCCTTTAATTTCTTTGGA, R:ATTCACCAGATCATGTGCCA	*Glyma.18g273600* -18 kb
	8	Ping et al. (2014)	F:AATTTGATGCACTTGATAACGA, R:TGACAAACACAAGAACTCACACA	*Glyma.18g273600* promoter
	9	Ping et al. (2014)	F:GAATCCACCATCACCAAACC, R:CAATGGCAACCCAGTAAGGT	*Glyma.18g273600* + 12 kb
*E1*	12	Xia et al. (2012)	F:TCAGATGAAAGGGAGCAGTGTCAAAAGAAGT, R:TCCGATCTCATCACCTTTCC	*Glyma.06g207800* exon 1
	13	Tsubokura et al. (2013b)	F:CACTCAAATTAAGCCCTTTCA, R:TTCATCTCCTCTTCATTTTTGTTG	*Glyma.06g207800* exon 1
	37	Tsubokura et al. (2013b)	F:CCGTTTGATTGGTTTTTGGT, R1:CCCTTCAGTTTCTGCAGCTC, R2:GAGAAGACAAACAATTCGAG	*Glyma.06g207800* exon 1
*E2*	15	Watanabe et al. (2011)	F:GAAGCCCATCAGAGGCATGTCTTATT, R:AAGCCTATGCCAGCTAGGTATTT	*Glyma.10g221500* exon 10
	17	Tsubokura et al. (2013b)	F:TGTTGATATTACATGCACATGCAT, R:GGCAGTTTCACCTTCTTAGC	*Glyma.10g221500* intron 8
*E3*	18	Tsubokura et al. (2013b)	F:TGGAGGGTATTGGATGATGC, R1:CTAAGTCCGCCTCTGGTTTCAG, R2:CGGTCAAGAGCCAACATGAG, R3:GTCCTATACAATTCTTTACGACG	*Glyma.19g224200* intron 3
	19	Tsubokura et al. (2013b)	F:TTGCATGAAGTTTTGGTTGC, R:CAACTGAACTGAAGACCCACAA	*Glyma.19g224200* exon 3
	20	Xu et al. (2013)	F:GGGATAGTTCTGATGCTGTTCAA, R:CCTTGTATCGATAGCATATGTGCT	*Glyma.19g224200* exon 1
	21	Xu et al. (2013)	F:GTTGAAGAGAAGATCACAACA, R:GATGAACTAATTTCCCTAACTGCA	*Glyma.19g224200* exon 3
*E4*	22	Liu et al. (2008)	F:AGACGTAGTGCTAGGGCTAT, R1:GCATCTCGCATCACCAGATCA, R2:GCTCATCCCTTCGAATTCAG	*Glyma.20g090000* exon 2
	23, 24	Tsubokura et al. (2013a)	F:CTTAATAAAGCCATGACTGGTTTG, R:CTTGAGTTTCAATGAGGTTTCAAC	*Glyma.20g090000* exon 3
	25	Tsubokura et al. (2013a)	F:CCCAGACACTCTTGTGTGAT, R:CCATACTCTCGGTATCTTTG	*Glyma.20g090000* exon 2
	26	Tsubokura et al. (2013a)	F:CACCCTAGGAGTTGTGTTGTT, R:GCGGTTCTGTACAATTGCCTGATA	*Glyma.20g090000* exon 3
*E7*	27	Molnar et al. (2003)	F:ACCTCATTTTGGCATAAA, R:TTGGAAAACAAGTAATAATAACA	unknown gene
*E9*	28	Kong et al. (2014)	F:GGGAAACTGAAAACTTAGGG, R:AAAGGAGCAGCAAAACGCTA	*Glyma.16g150700* promoter
	29	Zhao et al. (2016)	F:GGAATCGAGGCTATTGACTA, R:CTTCCACTAGGCATGGGATA	*Glyma.16g150700* 5’UTR
	30	Zhao et al. (2016)	F:TTCAAACAATCTCATAATTATGAGT, R:TAATAGTAGTATGGATGGTCAAA	*Glyma.16g150700* intron1
	31	Zhao et al. (2016)	F1:GCTCTCTCTCTTCCACTCTCTAGATGG, F2:ACCCTCTCAAGTGGACATGT. R:CTAGGTGCATCGGGATCAAC	*Glyma.16g150700* intron1
*E10*	34	Samanfar et al. (2017)	F:CAACCACCTTCACGTTGACA, R:ATTGTGCATGCTCCAAGATG	*Glyma.08g363100* exon 3
	35	Samanfar et al. (2017)	F:GAACTCGTGAAGGGATCCAA, R:GAGCAAGATAGGCCAAAAGC	*Glyma.08g363100* 5’UTR
*qPHD1*	*38*	Kim et al. (2020); Lee et al. (2017)	F:CAAATCCCTAGTCCAATCTTAGCCCAAG, R:ACATAACCATAAACACTTGCACTTCCT	*Glyma.16g141600* promoter

**Table 2:** Detection methods, recognized alleles, and optimized PCR and electrophoresis conditions for molecular markers tagging growth determination, early maturity, and pod shattering genes

Gene	No	Polymorphism type	Detection method	Recognized alleles	*T* °C^a^	Gel %^b^	Gel type	Samples per gel^c^
*Dt1*	1	SNP	CAPS, *Nde*I	*dt1-b*: 410 bp, other: 193 + 217 bp	54	2	ST	96
	2	INDEL	PCR	*dt1-b*: 319 bp, other: 325 bp	56	3	HR	48
	3	INDEL + SNP	CAPS, *Afl*II	*dt1-b*: 319 bp, other: 201 + 124 bp	56	3	ST	96
	4	SNP	dCAPS, *Xba*I	*dt1-ab*: 176 bp, other: 21 + 155 bp	60	3	ST	96
	5	SNP	CAPS, *Hin*dIII	*dt1-bb*: 215 + 593 bp, other: 808 bp	57	2	ST	96
	6	SNP	CAPS, *Acc*I	*dt1-tb*: 70 + 738 bp, other: 70 + 92 + 646 bp	57	2	ST	96
	36	SNP	CAPS, *Ava*II	*dt1-ta*: 326 bp, other: 229 + 97 bp	56	2	ST	96
*Dt2*	7	SSR	PCR	*dt2*: *280 bp*, *Dt2*: other products	56	3	HR	48
	8	SSR	PCR	*dt2*: *221 bp*, *Dt2*: other products	56	3	HR	48
	9	SSR	PCR	*dt2*: *202 bp*, *Dt2*: other products	57	3	HR	48
*E1*	12	SNP	dCAPS, *Taq*I	*e1-as*: 31 + 413 bp, *E1*/*e1-fs*/*e1-nl*: 443 or 444 bp,	55	3	ST	96
	13	SNP	CAPS, *Hin*fI	*E1*/*e1-as*: 36 + 186 bp, *e1-fs*: 36, 46 + 136 bp, *e1-nl*: unspecific products	52	2	ST	96
	37	INDEL	PCR	*E1/e1-as/e1-fs:* 841/840 bp, *e1-re*: 592 bp, *e1-nl*: no product	54	2	ST	96
*E2*	15	SNP	dCAPS, *Dra*I	*E2*: 130 bp, *e2*: 27 + 103 bp	58	2	ST	96
	17	INDEL	PCR	*E2-in*: 548 bp, *E2-dl*: 512 bp / e2 ?	57	2	ST	96
*E3*	18	INDEL	PCR	*E3-Misuzudaizu*: 1339 bp, *E3-Harosoy*: 558 bp *E3-Moshidougong*: 558 bp, *e3-tr*: 275 bp	57	2	ST	96
	19	SNP	CAPS, *Mse*I	*E3-Moshidougong* – 101 + 223 bp, other: 324 bp	57	2	ST	96
	20	SNP	CAPS, *Ale*I	*e3-fs*: 759 bp, other 206 + 552 bp	60	2	ST	96
	21	SNP	dCAPS, *Mfe*I	*e3-ns*: 23 + 140 bp, other: 163 bp	54	3	ST	96
*E4*	22	INDEL	PCR	*e4-SORE-1*: 837 bp, other: 1229 bp	56	2	ST	96
	23	SNP	CAPS, *Afl*II	*e4-kam*: 208 + 286 bp*,* other: 494 bp	56	2	ST	96
	24	SNP	CAPS, *Bsp*HI	*e4-kes*: 95 + 399 bp, other: 494 bp	56	2	ST	96
	25	SNP	CAPS, *Sac*I	*e4-oto*: 96 + 439 bp, other: 535 bp	53	2	ST	96
	26	SNP	dCAPS, *Eco*Rv	*e4-tsu*: 23 + 332 bp, other: 355 bp	56	2	ST	96
*E7*	27	SSR	PCR	*E7-168 bp, e7*: other products	54	3	HR	48
*E9*	28	INDEL	PCR	*E9Harosoy:* 187 bp, other: 230 bp	54	3	ST	96
	29	INDEL	PCR	*E9indel10*: 134 bp, other: 144 bp	54	3	HR	96
	30	SNP	dCAPS, *HinfI*	*E9SNP#17*: 138 bp, other: 160 bp	52	2	ST	96
	31	SORE-1 INDEL	PCR	*e9Toyomusume*: 306 bp, *E9*: 440 bp	58	2	ST	96
*E10*	34	SNP	CAPS, *MaeI BfaI*	*e10_exonSNP*: 116 bp, *E10*: 78 + 38 bp	56	2	ST	96
	35	SNP	dCAPS, *TaqI*	*E10*: 110 bp, *e10_3UTRSNP*: 82 + 28 bp	57	3	ST	96
*qPHD1*	38	SNP	dCAPS, *StyI*	*KSS-SNP5(G*): 24 + 51 bp*, KSS-SNP5(A)*: 75 bp	54	3	ST	96

Agarose type: *ST* standard (wide range), *HR* high resolution (3:1)

^a^Annealing temperature of PCR primers

^b^Agarose gel concentration

^c^Number of samples loaded on a 20 × 25 cm gel tray for a single run

### Genotyping soybean diversity panel for early maturity, growth determination, and pod shattering genes

The set of markers used for soybean genotyping included 12 CAPS, eight dCAPS, eight typical PCR INDEL and four PCR SSR markers. Young 6 week-old leaves were collected from plants cultivated in greenhouse. Plant tissue frozen under liquid nitrogen (50–100 mg) was homogenized using TissueLyser II (Qiagen, Hilden, Germany) and two stainless steel beads (ø 5 mm) placed in a 2-mL tube (Eppendorf, Hamburg, Germany). DNA isolation was performed using Maxwell RSC PureFood GMO and Authentication Kit (Promega, Madison, USA) and standard protocol implemented in Maxwell 16 DNA isolation system (Promega). DNA concentration and purity were measured using NanoDrop 2000 (ThermoFisher Scientific, Waltham, USA) and A260/A280 ratio. All PCR reactions were performed using GoTaq® Flexi DNA Polymerase (Promega), Labcycler Gradient thermal cycler (Sensoquest, Göttingen, Germany), 96-well PCR plates (4titude, Wotton, Surrey, UK), and standard tips (Neptune Scientific, San Diego, USA). Restriction enzymes were derived from New England Biolabs (Ipswich, USA) and Thermo Fisher Scientific (Waltham, USA). Restriction products or PCR amplicons were separated by agarose gel electrophoresis using standard (Wide Range, Serva, Heidelberg, Germany) or high-resolution agarose (3:1, Serva) with the agarose concentration (1–3%) adjusted to follow the size of the expected digestion products. Electrophoresis buffer and gels were prepared using standard Tris–Borate-EDTA (Serva). Visualization of electrophoresis result was performed by in-gel SYBRSafe (ThermoFisher Scientific) staining and UV transillumination Essential system (Uvitec, Cambridge, United Kingdom), with F-520 (amber) photographic filter (Uvitec). Two biological replicates were analyzed per line. Marker alleles associated with early maturity, determinate growth, and non-shattering pods were assigned “1,” opposite alleles were assigned “2,” whereas heterozygotes “1.5.”

### Phenotyping soybean diversity panel for phenology and yield-related traits

The field experiment was carried out in 2018–2020 at the Dłoń Agricultural Research Station, the Poznań University of Life Sciences, Poland (51° 41′ 29″ N, 17° 4′ 34″). Soybean diversity panel was sown at the following dates: 20 Apr 2018, 24 Apr 2019 and 22 Apr 2020 at 2 m^2^ field plots (two replications) with 50-cm row spacing and density of 60 seeds per 1 m^2^. The fertilizer was used according to the conventional farming practices in this area (N 30 kg∙ha^−1^, P 80 kg∙ha^−1^, K 120 kg∙ha^−1^). Meteorological conditions were measured in field trails according to the WMO standards using a Vantage Vue 6357 UE 9 meteorological station (Davis Instruments, USA). Observed values (monthly and daily mean values) are provided in the Supplementary Table S2.

Based on the whole plot observations, plant phenology and growth habit (determinate, semi-determinate, and indeterminate) were evaluated. Phenology observations included number of days from sowing to flowering of 50% plants in a plot and number of days from sowing to pod harvest maturity of 50% plants in a plot. Harvest maturity of a plant was considered when a R8 reproductive stage was reached (Fehr and Caviness 1977). Based on the five typical even representative plants per each plot, the following measurements and calculations were done: plant height, number of lateral branches, first pod height, number of pods per plant, number of seeds per plant, number of seeds per pod, seed weight per plant, and thousand grain weight (TGW). Apart from plant phenotypic traits, five environmental traits were calculated using daily meteorological data measured at observation site during experiments: the number growing degree days (GDDs) from sowing to flowering and from sowing to maturity as well as amount of precipitation (mm) from sowing to flowering, from sowing to maturity, and between flowering and maturity. For GDD calculations, base temperature of 10 °C was used (Karges et al. 2022).

### Statistical analysis and data visualization

All statistical analyses and data visualizations were preferred in R software (R Core Team 2013, Vienna, Austria) using packages ggplot2, dplyr, and tidyr. Spearman’s rank correlation between allelic phases and analyzed traits was calculated using standard function “cor.test” which reports both correlation and corresponding *p* value. Spearman’s rho statistic was used to estimate a rank-based measure of association. An approximation of the exact null distribution of Spearman’s rank correlation statistics was made using the AS89 algorithm (Hollander and Wolfe 1973). Custom R script was built to loop for all combinations of marker and trait correlation. The Pearson correlation between analyzed traits and corresponding *p* values were calculated using “rcorr” function from Hmisc packages (https://hbiostat.org/R/Hmisc/). One-way ANOVA test was applied to estimate broad sense heritability. The genetic variance (${\sigma }_{G}^{2}$) was calculated as $\frac{{MS}_{G}-{MS}_{e}}{r}$ and residual variance (${\sigma }_{e}^{2}$) as ${MS}_{e}$. Finally, the heritability was calculated using the formula ${H}^{2}=\frac{{\sigma }_{G}^{2}}{{\sigma }_{G}^{2}+{\sigma }_{e}^{2}/r}$.

## Results

### Implementation of the PCR array tagging growth determination, early maturity, and pod-shattering genes

The array of 32 molecular markers tagging early maturity (*E1*, *E2*, *E3*, *E4*, *E7*, *E9*, and *E10*), growth determination (*Dt1* and *Dt2*), and pod-shattering (*qPHD1*) genes (Kim et al. 2020; Kong et al. 2014; Lee et al. 2017; Liu et al. 2008, 2010; Molnar et al. 2003; Ping et al. 2014; Samanfar et al. 2017; Tian et al. 2010; Tsubokura et al. 2013a, 2013b; Watanabe et al. 2009, 2011; Xia et al. 2012; Xu et al. 2013; Zhao et al. 2016) was implemented in this study (Table 2). Apart from using published primers, we designed new markers for two *Dt1* alleles (Liu et al. 2010; Tian et al. 2010) and one new marker for the pod shattering (*qPHD1*) gene (Kim et al. 2020; Lee et al. 2017) that have not yet been supplied with PCR-based genotyping tool. Optimization procedure was effective for all markers (Supplementary Fig. 1), enabling routine screening of 96 samples (27 markers) or 48 samples (five markers) per single agarose gel. Twenty-six markers could be resolved using cost-efficient molecular biology grade agarose gel, in concentration of 2% (19 markers) or 3% (seven markers), whereas six markers required high-resolution 3:1 agarose gel in concentration of 3%. Alleles could be recognized with high confidence for all markers except those tagging the *Dt2* gene due to the presence in soybean diversity panel of three to four homozygous alleles per marker with product length similar to the target amplicons (Supplementary Fig. 1). Therefore, results of marker screening for the *Dt2* gene were not included in the phenotype-genotype association study.

### Genotyping of soybean diversity panel with PCR array

Genotyping with the array of 29 markers (i.e., all markers from Table 2 except those for *Dt2*) revealed the presence of complex pattern of allele distribution in soybean diversity panel. Forty-one allelic combinations were identified when simple classification to recessive or dominant alleles was applied, whereas when diversity among dominant or recessive alleles was taken into account, the number of identified combinations raised to 98. Minor allele frequency varied from 0.5% (*e9*) to 39% (*e4*). Taking into consideration growth determination gene *Dt1* (*Glyma.19g194300*), dominant (wild, indeterminate) *Dt1* and *Dt1-b* alleles were found in 79 and 78 accessions, respectively, whereas recessive (domesticated, determinate) alleles *dt1-tb* and *dt-ab* in 26 and 21 accessions, respectively. Recessive (domesticated, early) alleles of an early maturity gene *E1* (*Glyma.06g207800*) were identified in 170 accessions, namely, *e1-as* in 99, *e1-nl* in 65, *e1-fs* in four and heterozygous *e1-as*/*e1-nl* in two accessions. One hundred eighty-nine accessions yielded a recessive (domesticated, early) allele of an early maturity gene *E2* (*Glyma.10g221500*). An early maturity gene *E3* (*Glyma.19g224200*) revealed the highest diversity of present alleles among studied genes, with three dominant (wild, late) alleles (*E3-Harosoy*, *E3-Misuzudaizu*, and *E3-Moshidougong* found in 53, 3, and 1 accession, respectively) and three recessive (domesticated, early) alleles (*e3-tr*, *e3-fs*, *e3-ns*, and *e3-tr*/*e3-fs* identified in 88, 39, 19, and 1 accession, respectively). Eighty accessions were revealed to carry recessive (domesticated, early) alleles of an early maturity gene *E4* (*Glyma.20g090000*): *e4-SORE1*, *e4-kes*, and *e4-kam* (70, 8 and 2 accessions, respectively). Recessive (domesticated, early) alleles of early maturity *E7* (unknown) and *E10* (*Glyma.08g363100*) genes were identified in 141 and two accessions, a recessive (domesticated for low-latitude regions, late) allele of an early maturity gene *E9* (*Glyma.16g150700*) in one accession, whereas a domesticated (non-shattering) allele *KSS-SNP5(A)* of a pod-shattering *qPHD1* (*Glyma.16g141600*) gene in 143 accessions. Allelic composition of earliness genes clearly influenced the length of the vegetation period. In the group of the 31 earliest genotypes, with a vegetation period of 126.7–137.7 days, the majority (21) of the varieties had a set of four recessive alleles in the four main *E1*–*E4* loci. Of these 21 genotypes, only six were characterized by an indeterminate growth type—the *Dt1* and *Dt1-b* alleles. On the other hand, genotypes with four or three dominant alleles matured the latest on October and November. All Polish contemporary cultivars (except Erica, which had the *E3* allele) had four recessive alleles and a similar, short vegetation period (133.7–138.3 days). Also almost all breeding lines, 14 out of 16, had four recessive alleles. The old Polish cultivars Warszawska and Złotka had a dominant *E1* allele. Results of soybean diversity panel genotyping with PCR marker array are provided in the Supplementary Table S3.

### Variability of phenology and yield-related traits in soybean diversity panel

Phenotyping of phenology-, height-, and yield-related traits in 2018–2020 years revealed significant differences between genotypes. Taking into account mean values calculated for 3 years, relative variability was the highest for the number of pods per plant, first pod height, and the number of seeds per plant, whereas the lowest for the number of days to maturity and the number of seeds per pod. Thus, the number of days to flowering ranged from 51.7 ± 7.8 to 105.7 ± 10.8, the number of days to maturity from 126.7 ± 8.4 to 199.3 ± 12.7, plant height from 21.5 ± 3.0 to 110.1 ± 7.7, first pod height from 4.4 ± 0.4 to 26.8 ± 5.7, the number of lateral shoots from 1.2 ± 0.9 to 5.3 ± 1.2, the number of pods per plant from 15.6 ± 6.9 to 127.6 ± 69.9, the number of seeds per plant from 22.4 ± 10.8 to 213.5 ± 100.9, the number of seeds per pod from 1.3 ± 0.1 to 2.2 ± 0.2, seed yield per plant from 6.3 ± 3.4 to 38.0 ± 11.4, and thousand grain weight from 130.3 ± 0.9 to 374.2 ± 37.2. Detailed phenotypic observations are provided in the Supplementary Table S4.

Broad-sense heritability was the highest for plant height, thousand grain weight, the number of days to maturity, and first pod height (range from 0.84 to 0.88); the number of days to flowering revealed moderate heritability value (0.52), whereas the lowest heritability was revealed for seed yield per plant and number of seeds per plant (0.04 and 0.19, respectively) (Table 3). These results demonstrated high potential of artificial selection towards desired plant height, phenology, and seed size during soybean breeding in Poland.

**Table 3:** Mean, minimum, and maximum values observed for phenology and yield-related traits during field experiments and broad sense heritability

Year	2018			2019			2020			2018–2020
Trait	Mean	Min	Max	Mean	Min	Max	Mean	Min	Max	Heritability
Days to flowering	53.6	41	91	62.6	50	109	75.1	56	127	0.52
Days to maturity	150.8	116	193	152.6	118	188	163.0	133	217	0.84
Plant height (cm)	68.6	22	127	64.1	18	116	63.1	15	110	0.88
First pod height (cm)	10.9	4	25	10.8	3	33	9.7	3	28	0.84
Number of lateral shoots	2.3	0	8	3.0	1	8	3.3	1	7	0.23
Number of pods per plant	42.8	4	226	50.2	13	107	57.8	10	141	0.26
Number of seeds per plant	72.3	5	356	94.1	21	207	115.8	16	305	0.19
Number of seeds per pod	1.7	1	2	1.9	1	2	2.0	1	3	0.34
Seed yield per plant (g)	14.6	1	49	19.6	5	54	25.5	4	63	0.04
Thousand grain weight (g)	210.4	103	419	211.1	93	342	224.9	129	405	0.86

**Fig. 1:**
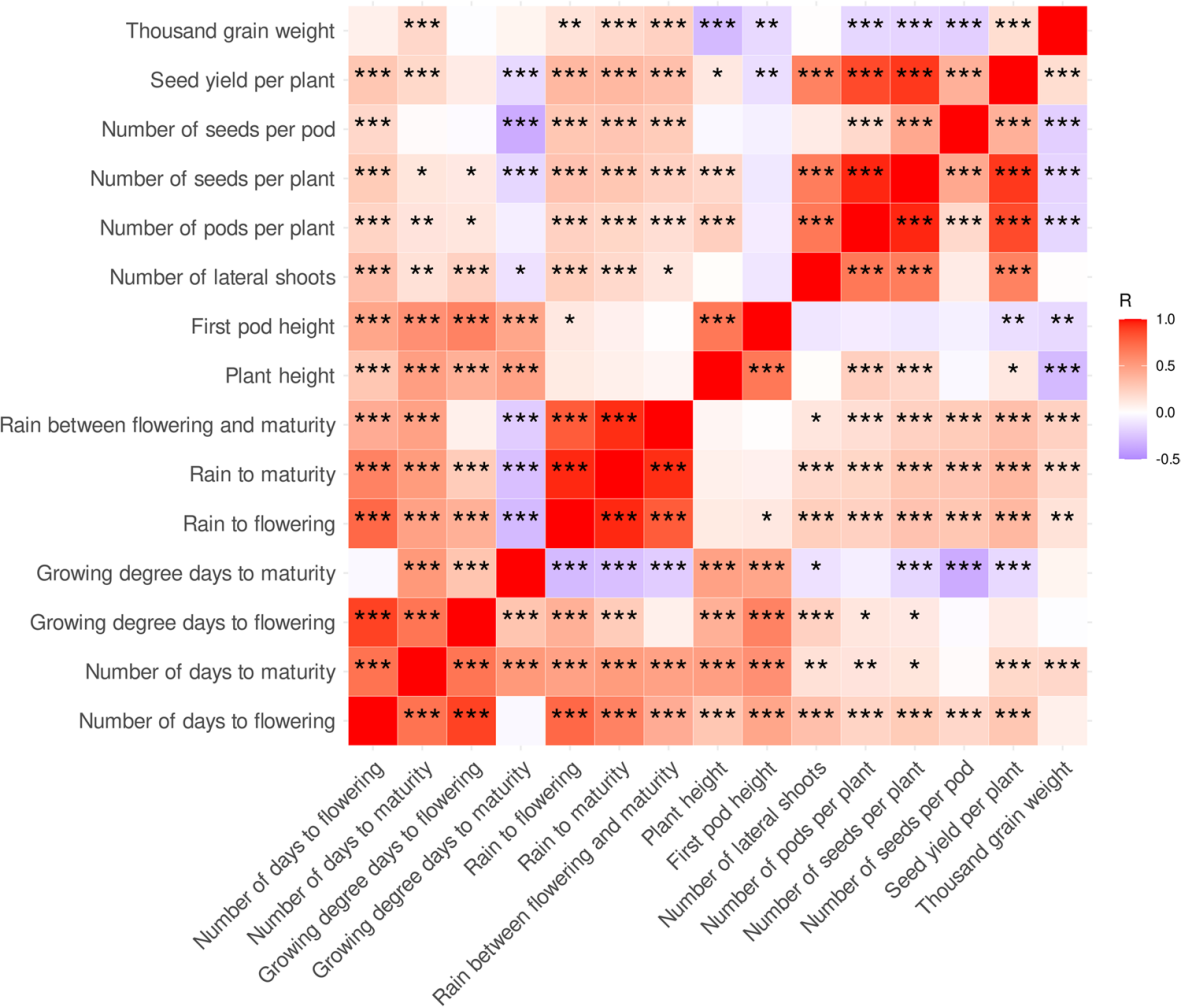
Correlation heatmap reporting Spearman’s rank correlation coefficients for each trait vs trait comparison. Observations were performed during 2018, 2019, and 2020 growing seasons in Dłoń Agricultural Research Station, the Poznań University of Life Sciences, Poland (51° 41′ 29″ N, 17° 4′ 34″). The bar below the heatmap indicates the color legend of correlation coefficients. Asterisk (*) indicates significant correlations in the following scheme: ***p < 0.0001; **0.0001 ≤ *p* < 0.001; *0.001 ≤ *p* ≤ 0.01

Observed phenotypic traits revealed remarkable direct and indirect relationships in studied environmental conditions (Fig. 1, Supplementary Table S5). The most significant positive correlations were found between the number of seeds per plant and the number of pods per plant (0.95), as well as between these two traits and seed yield per plant (0.84 and 0.80). Moreover, high correlations were also identified between trait related with plant phenology (the number of days to flowering and maturity), plant height, and first pod height (from 0.57 to 0.78). On the other hand, thousand grain weight revealed moderate negative correlation with plant height, the number of pods and seeds per plant, and the number of seeds per pod (from − 0.36 to − 0.31). All studied traits revealed significant differences between observed values in 2018 and 2020 years, six traits between 2018 and 2019, whereas seven traits between 2019 and 2020 (Supplementary Table S6). To evaluate the influence of temperature and precipitation during growing season on soybean phenotypic traits, we supplemented our correlation analysis with the following environmental traits that were calculated using daily meteorological data measured at observation site during experiments: GDDs from sowing to flowering and from sowing to maturity as well as amount of precipitation from sowing to flowering, from sowing to maturity, and between flowering and maturity. Yield-related traits (the number of seeds per plant, the number of seeds per pod, and seed yield per plant) revealed significant positive correlations with amount of precipitation from sowing to flowering (0.29–0.36), from sowing to maturity (0.29–0.36), and from flowering to maturity (0.24–0.33). Thousand grain weight revealed the highest positive correlation with amount of precipitation from flowering to maturity (0.23).

To analyze differences between years in different phenology groups, germplasm diversity panel was divided into three categories: early (66 accessions characterized by mean days to maturity from 126.7 to 145), intermediate (65 accessions with mean days to maturity between 145.3 and 160), and late (70 accessions with mean maturity between 160.3 and 199.3 days). Remarkably significant differences between years were observed for three major yield-related traits: the number of seeds per plant, thousand grain weight (g), and seed yield per plant (g) (< 0.00001). In general, the lowest values were observed in 2018, whereas the highest in 2020 (Table 4). It should be noted that in the growing season enabling the best soybean performance (2020), moderate and late flowering germplasm significantly out-yielded early accessions (*P* < 0.0001), producing also significantly higher number of seeds per plant (*P* < 0.002). Late lines developed also in that year significantly larger seeds than the early genotypes (*P* = 0.008).

**Table 4:** Mean values of yield-related traits calculated for different phenology groups and significance of differences between years

Group	Year	Number of seeds per plant	Thousand grain weight (g)	Seed yield per plant (g)
Early	2018	65.8	198.8	13.0
	2019	99.2	201.2	19.8
	2020	97.7	215.2	20.5
	2018 vs 2019	***	NS	***
	2019 vs 2020	NS	*	NS
	2018 vs 2020	***	*	***
Intermediate	2018	73.7	212.3	14.5
	2019	93.7	214.6	19.9
	2020	128.2	222.7	28.1
	2018/2019	**	NS	***
	2019/2020	***	NS	***
	2018/2020	***	NS	***
Late	2018	77.2	219.6	16.3
	2019	89.7	217.2	19.1
	2020	121.4	236.1	27.8
	2018/2019	0.06	0.74	*
	2019/2020	***	*	***
	2018/2020	***	*	***
Intermediate vs early	2020	***	NS	***
Late vs early	2020	**	**	***

*NS* non-significant

^***^p < 0.001; **0.001 ≤ *p* < 0.01; *0.01 ≤ *p* ≤ 0.05

### Applicability of PCR array for molecular selection of soybean towards cultivation in Central Europe

**Fig. 2:**
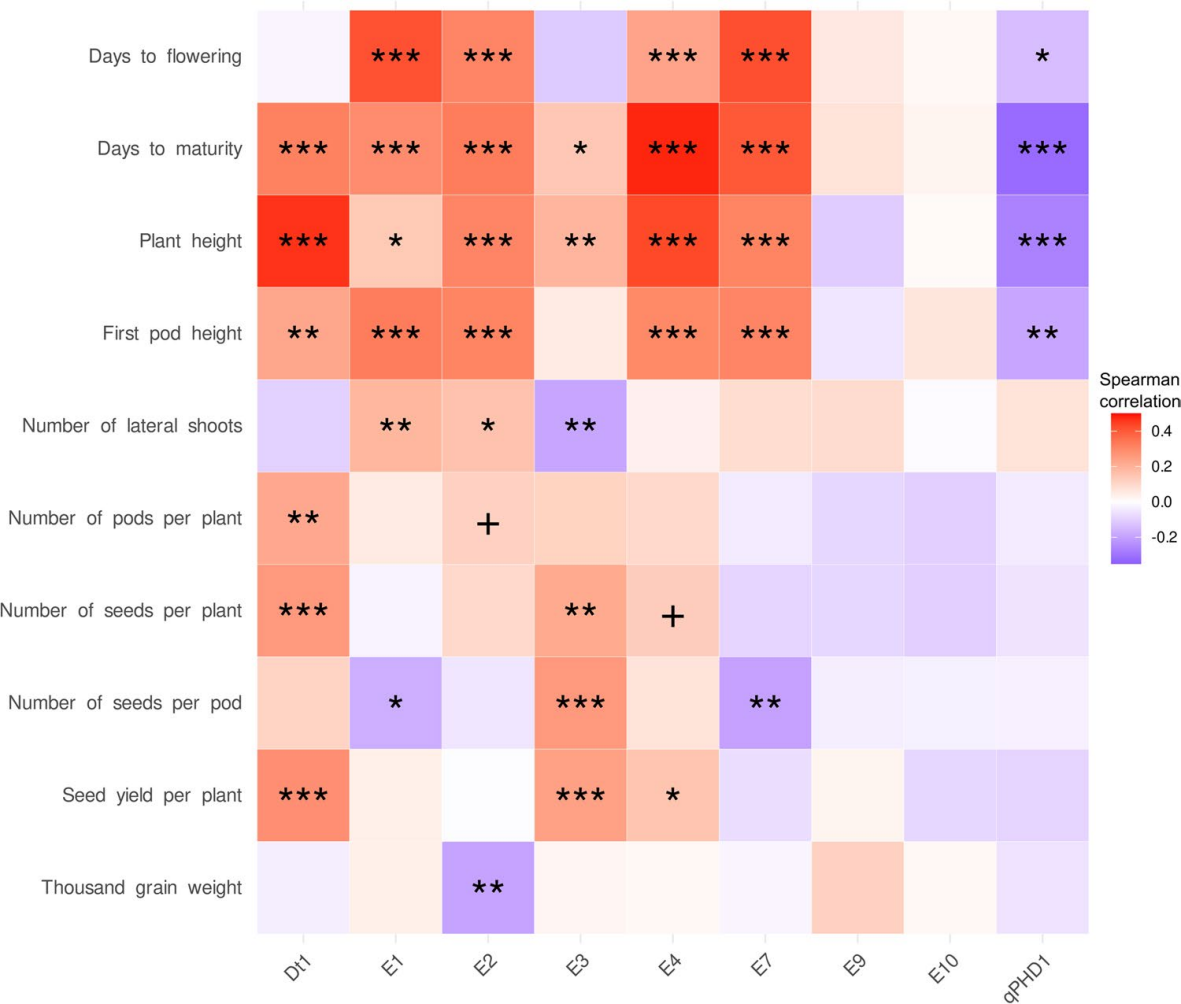
Correlation heatmap reporting Spearman’s rank correlation coefficients for each trait vs gene comparison. *E1*, *E2*, *E3*, *E4*, *E7*, *E9*, *E10*—early maturity genes, *qPHD1*—pod-shattering gene. Domesticated alleles were coded as 1, whereas wild alleles as 2. Observations were performed during 2018, 2019, and 2020 growing seasons in Dłoń Agricultural Research Station, the Poznań University of Life Sciences, Poland (51° 41′ 29″ N, 17° 4′ 34″). The bar below the heatmap indicates the color legend of correlation coefficients. Asterisk (*) indicates significant correlations in the following scheme: ***p < 0.001; **0.001 ≤ *p* < 0.01; *0.01 ≤ *p* ≤ 0.05; + 0.05 < *p* ≤ 0.1

All genes except *E9* and *E10* revealed significant correlations with at least three traits (Fig. 2, Supplementary Table S7). The lack of significant correlations for *E9* and *E10* genes results from very low frequency of minor alleles observed for these genes (below 1%). Genes *Dt1*, *E1*, *E2*, and *E3* revealed significant correlation with six traits, genes *E4* and *E7* with five traits, whereas a *qPHD1* gene with three traits.

Days to maturity and plant height revealed significant correlations with seven genes, first pod height with six genes, days to flowering with four genes, the number of lateral shoots, the number of seed per pod and seed yield per plant with three genes, the number of seeds per plant with two genes, whereas the number of pods per plant and thousand grain weight with one gene. The most remarkable correlations were those identified for days to flowering with *E7* and *E1* genes (0.42 and 0.41), days to maturity with *E4* and *E7* (0.48 and 0.40), and plant height with *Dt1* and *E4* (0.46 and 0.43).

**Fig. 3:**
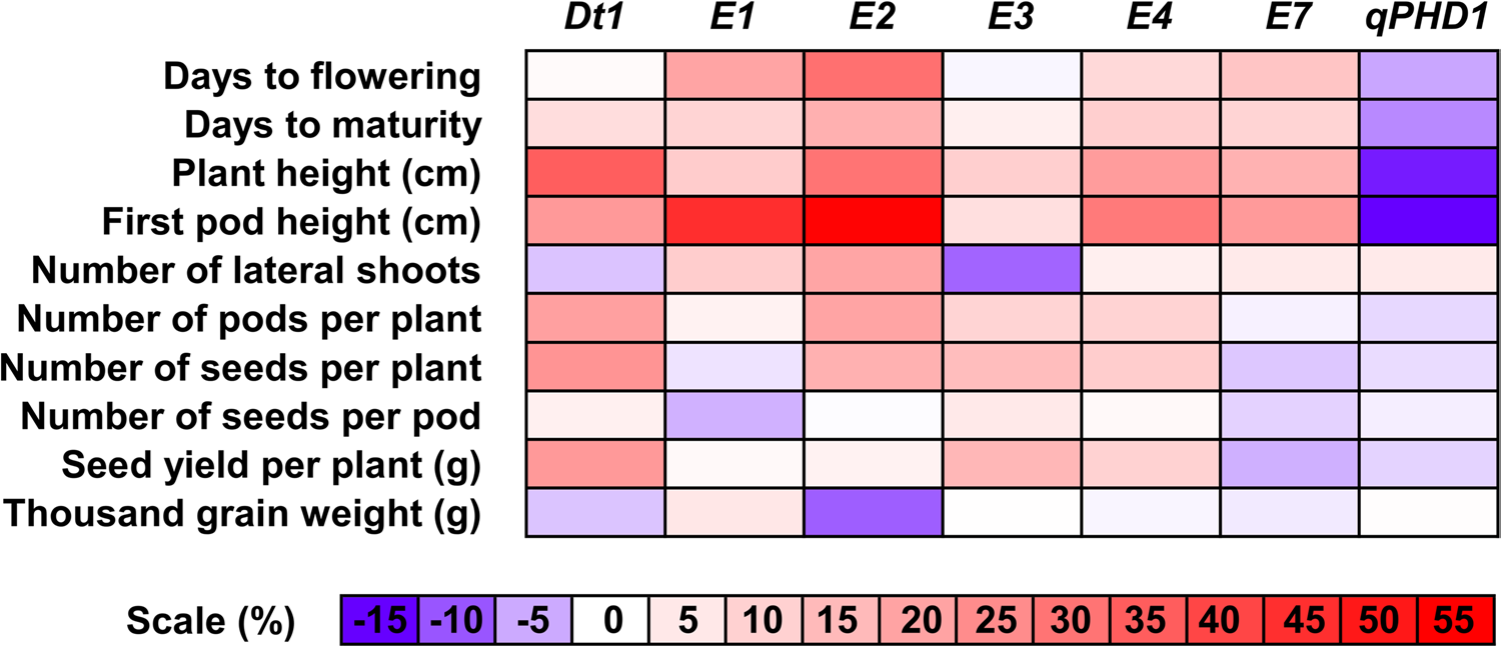
Effects of wild alleles of soybean growth determination (*Dt1*), early maturity (*E1*, *E2*, *E3*, *E4* and *E7*) and pod-shattering (*qPHD1*) genes on phenology, height, and yield-related traits. Observations were performed during 2018, 2019, and 2020 growing seasons in Dłoń Agricultural Research Station, the Poznań University of Life Sciences, Poland (51° 41′ 29″ N, 17° 4′ 34″). Color scale visualizes percentage change of mean values from 3 years between lines carrying domesticated and wild alleles

It should be emphasized that genes differed by direction of effects of wild alleles of studied traits. Growth determination (*Dt1*) and all early maturity genes except *E7* conferred increase of mean values of eight to nine traits (from 10 analyzed) as compared to domesticated alleles, whereas *qPHD1* had generally negative effect (Fig. 3). Taking into consideration means calculated for absolute values of percentage changes between lines carrying domesticated and wild alleles, the most influential were *E2* (20.1% of mean trait percentage change between opposite allele phases), *Dt1* (14.2%), *E1* (11.3%) and *E4* (10.5%) genes. The most influenced traits were first pod height (27.9% of mean percentage change), plant height (19.8%), days to flowering (11.2%), and number of seeds per plant (10.4%).

**Fig. 4:**
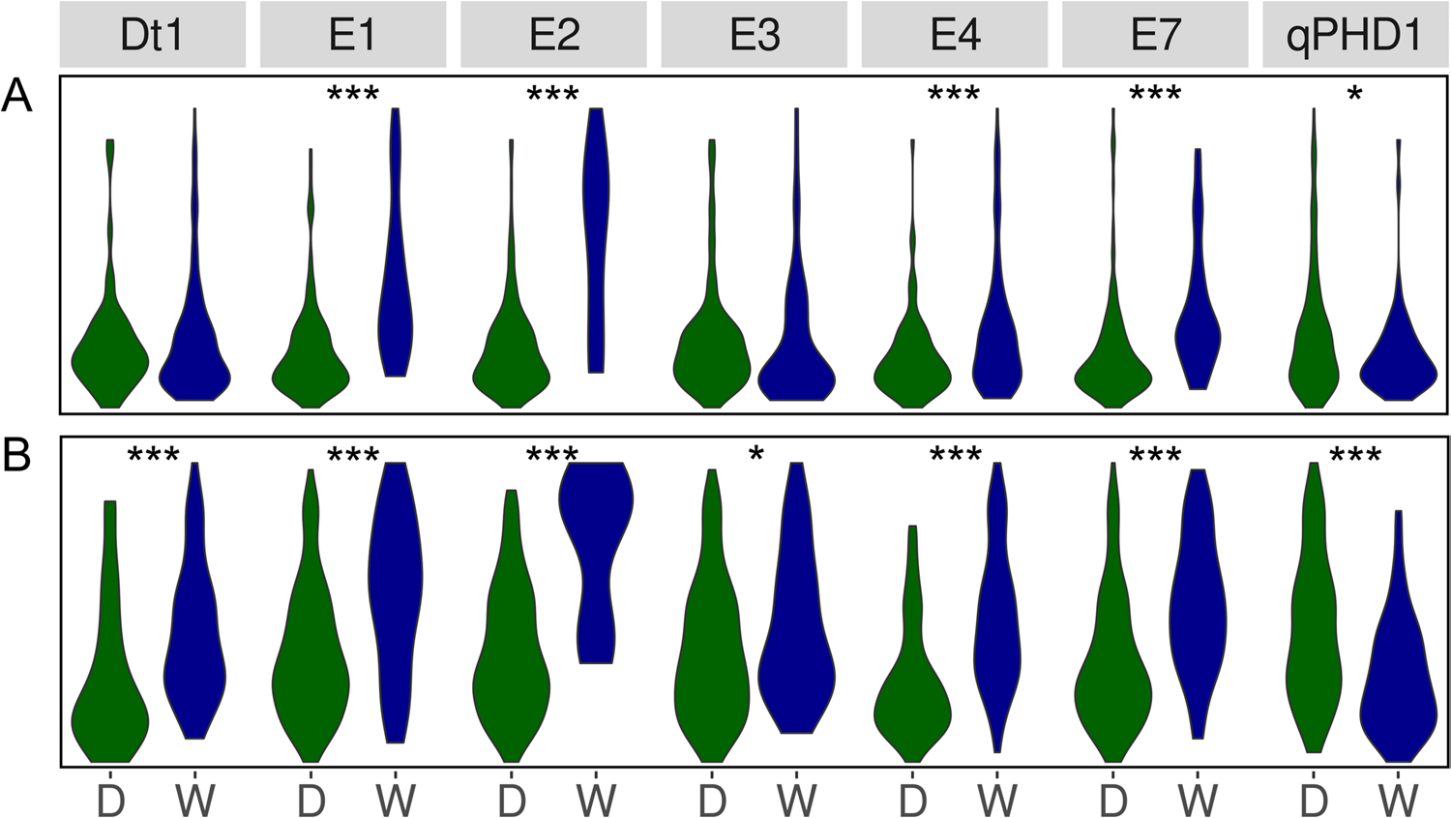
Allelic effects on days from sowing to flowering (**A**) and maturity (**B**) for soybean growth determination (*Dt1*), early maturity (*E1*, *E2*, *E3*, *E4*, and *E7*) and pod shattering (*qPHD1*) genes. D stands for a domesticated allele, whereas W for a wild allele. Observations were performed during 2018, 2019, and 2020 growing seasons in Dłoń Agricultural Research Station, the Poznań University of Life Sciences, Poland (51° 41′ 29″ N, 17° 4′ 34″). Asterisk (*) indicates significant correlations in the following scheme: ***p < 0.001; **0.001 ≤ *p* < 0.01; *0.01 ≤ *p* ≤ 0.05, + 0.05 < *p* ≤ 0.1

Taking into account direction of effects for wild alleles on phenology traits (Fig. 4), the highest percentage changes of mean values from 3 years were observed for *E2* (30.9% for days to flowering and 17.1% for days to maturity), *E1* (19.7% and 9.4%), *E7* (12.5% and 9.3%), and *E4* (8.2% and 10.4%). A wild allele of a *qPHD1* gene had moderate negative effect (− 5.5% and − 7.3%). Days to maturity revealed lower responsiveness than days to flowering, putatively due to longer exposure to variable environmental factors.

**Fig. 5:**
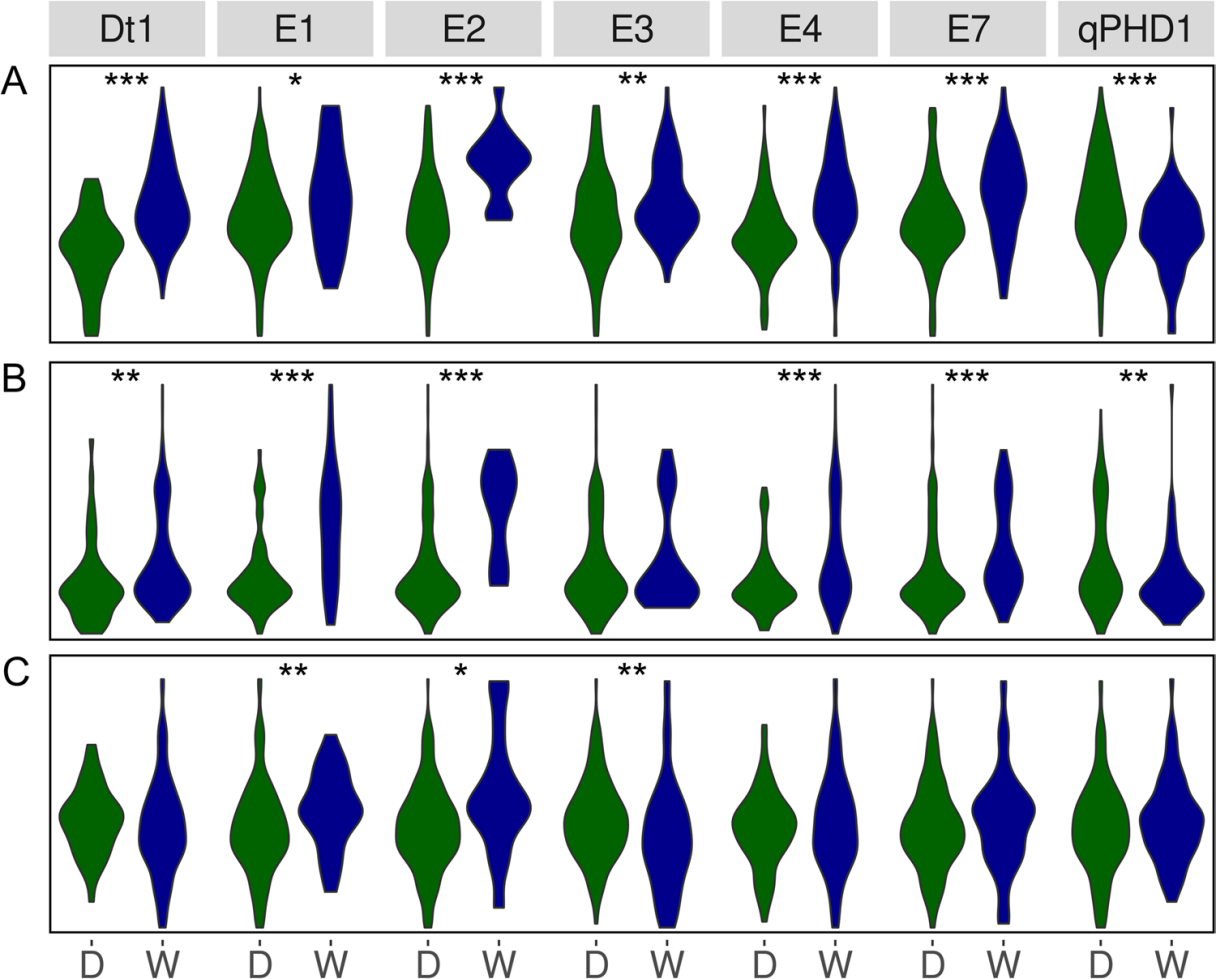
Allelic effects on plant height (**A**), first pod height (**B**) and the number of lateral shoots (**C**) for soybean growth determination (*Dt1*), early maturity (*E1*, *E2*, *E3*, *E4*, and *E7*), and pod shattering (*qPHD1*) genes. D stands for a domesticated allele, whereas W for a wild allele. Observations were performed during 2018, 2019, and 2020 growing seasons in Dłoń Agricultural Research Station, the Poznań University of Life Sciences, Poland (51° 41′ 29″ N, 17° 4′ 34″). Asterisk (*) indicates significant correlations in the following scheme: ***p < 0.001; **0.001 ≤ *p* < 0.01; *0.01 ≤ *p* ≤ 0.05, + 0.05 < *p* ≤ 0.1

From three morphological traits studied (plant height, first pod height and number of lateral shoots), the first two were highly influenced by allelic phases of studied genes (Fig. 5). The most remarkable effects were observed for first pod height and *E2* (54.5% of mean percentage change as compared to domesticated allele), *E1* (45.2%), *E4* (28.7%), *Dt1* (22.1%), and *E7* (21.9%) genes as well as for plant height and *Dt1* (34.8%), *E2* (30.0%), and *E4* (21.5%) genes. It should be noted that these two traits revealed the highest association among studied traits with a *qPHD1* gene, with relatively strong effect of wild allele (− 15.8% and − 14.2%, respectively). The third trait, number of lateral shoots, was rather only loosely associated with allelic phases of analyzed genes, except *E2* providing 19.4% of mean percentage change. Moderate effects were also revealed for *E1* (10.9%) and *E3* (− 9.6%); the latter was the only one significant negative effect observed for this trait and this marker.

**Fig. 6:**
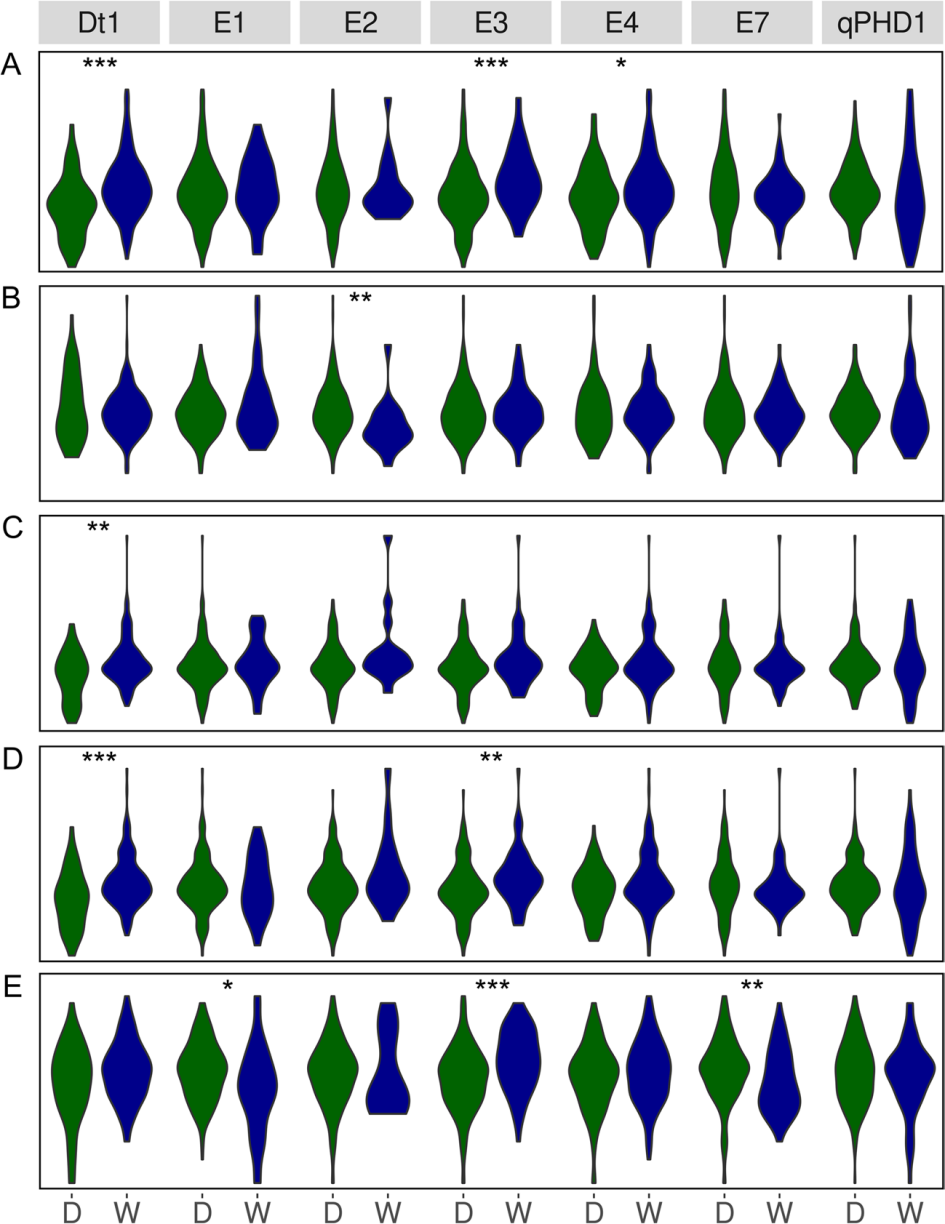
Allelic effects on seed yield per plant (**A**), thousand grain weight (**B**), the number of pods per plant (**C**), the number of seeds per plant (**D**), and the number of seeds per pod (**E**) for soybean growth determination (*Dt1*), early maturity (*E1*, *E2*, *E3*, *E4*, and *E7*) and pod-shattering (*qPHD1*) genes. D stands for a domesticated allele, whereas W for a wild allele. Observations were performed during 2018, 2019, and 2020 growing seasons in Dłoń Agricultural Research Station, the Poznań University of Life Sciences, Poland (51° 41′ 29″ N, 17° 4′ 34″). Asterisk (*) indicates significant correlations in the following scheme: ***p < 0.001; **0.001 ≤ *p* < 0.01; *0.01 ≤ *p* ≤ 0.05, + 0.05 < *p* ≤ 0.1

Major yield-related traits (seed yield per plant and thousand grain weight) revealed opposite allelic effects for significantly correlated genes (Fig. 6 A and B). Seed yield was positively influenced by wild alleles of *Dt1* (22.1% of mean percentage change as compared to domesticated allele), *E3* (15.6%), and *E4* (9.8%), whereas thousand grain weight was negatively influenced by *E2* (− 9.9%). Secondary yield-related traits (number of pods per plant, number of seeds per plant and number of seeds per pod) showed more coherent pattern of allelic effects, at least for the first two traits (Fig. 6C–E). The highest values of mean percentage change were observed for *Dt1* (20.5% and 23.2%, respectively), *E2* (19.7% and 17.0%), *E3* (9.3% and 14.3%), and *E4* (9.6% and 10.9%) genes. The last trait, number of seeds per pod, revealed lower values of percentage changes between allelic phases. The highest effects were calculated for *E3* (4.9%) and *E1* genes (− 4.8%).

## Discussion

The study showed a large genetic diversity of tested germplasm and demonstrated high applicability of PCR array for molecular selection of soybean towards adaptation to Polish agroclimatic conditions. By high correlations between phenotypic traits and allelic variants of *E1*, *E2*, *E3*, *E4*, and *E7* genes, the current research illustrated the strong effect of photoperiod on soybean performance at a given location that supports similar conclusion reached in the European mega-environment study of soybean germplasm (Kurasch et al. 2017). There is no doubt that the PCR array enabling determination of particular haplotypes at early maturity loci validated in the present study may facilitate selection of germplasm carrying optimal allelic combinations. Nevertheless, it should be taken into account that genetic control of phenology traits extend to additional pathways and soybean cultivars with the same haplotype at major *E* loci can differ in flowering and maturity dates (Kurasch et al. 2017).

Our research revealed also significant associations between an allelic phase of growth habit *Dt1* gene and plant phenology and yield-related traits (the number of pods and seeds per plant as well as overall seed yield). This observation converges with the recent findings that *Dt1* gene is targeted by SUPPRESSOR OF OVEREXPRESSION OF CONSTANS1 (SOC1/AGL20)–Dt2 complex, and functional divergence at SOC1 also affects soybean yield and latitudinal adaptation (Kou et al. 2022). It should be also noted that there is a direct cross-talk in soybean between flowering time and *Dt1* shoot determinacy driven by the interaction between FT5a and Dt1-APETALA1 feedback loop during long-day photoperiod (Yue et al. 2021). Therefore, selection for desired *Dt1* genotype (indeterminate) would prioritize delayed flowering under long photoperiod as observed in our study. The same statement can be concluded for a pod-shattering *qPHD1* gene because desired non-shattering allele is associated with delayed flowering. Currently, we do not have knowledge if this association was artificially introduced by soybean breeding under neutral photoperiod or if there is any functional link between these two traits. Since the vast majority of varieties and breeding lines bred in Poland has four recessive alleles and is very early, as a result of significant climate warming, they mature even earlier and therefore have a low yield compared to varieties with a longer vegetation period. According to the presented work, Polish breeders should introduce the *E3* or *E4* alleles, or maybe both, into breeding materials. Such selection is now possible using the PCR array. After development of validated markers for the *Dt2* gene, it will be necessary to check whether the indeterminate or semi-determinate type of growth is more profitable in local environmental conditions.

To summarize, the arrangement of alleles associated with the highest yield in studied environment encompassed dominant alleles for the *Dt1*, *E3*, and *E4* genes and the recessive allele for the *qPHD1* gene. Since this arrangement simultaneously determines the considerable height of plants and the late maturity date, it may be unfavorable in northern regions (i.e., above 54°N).

**Fig. 7:**
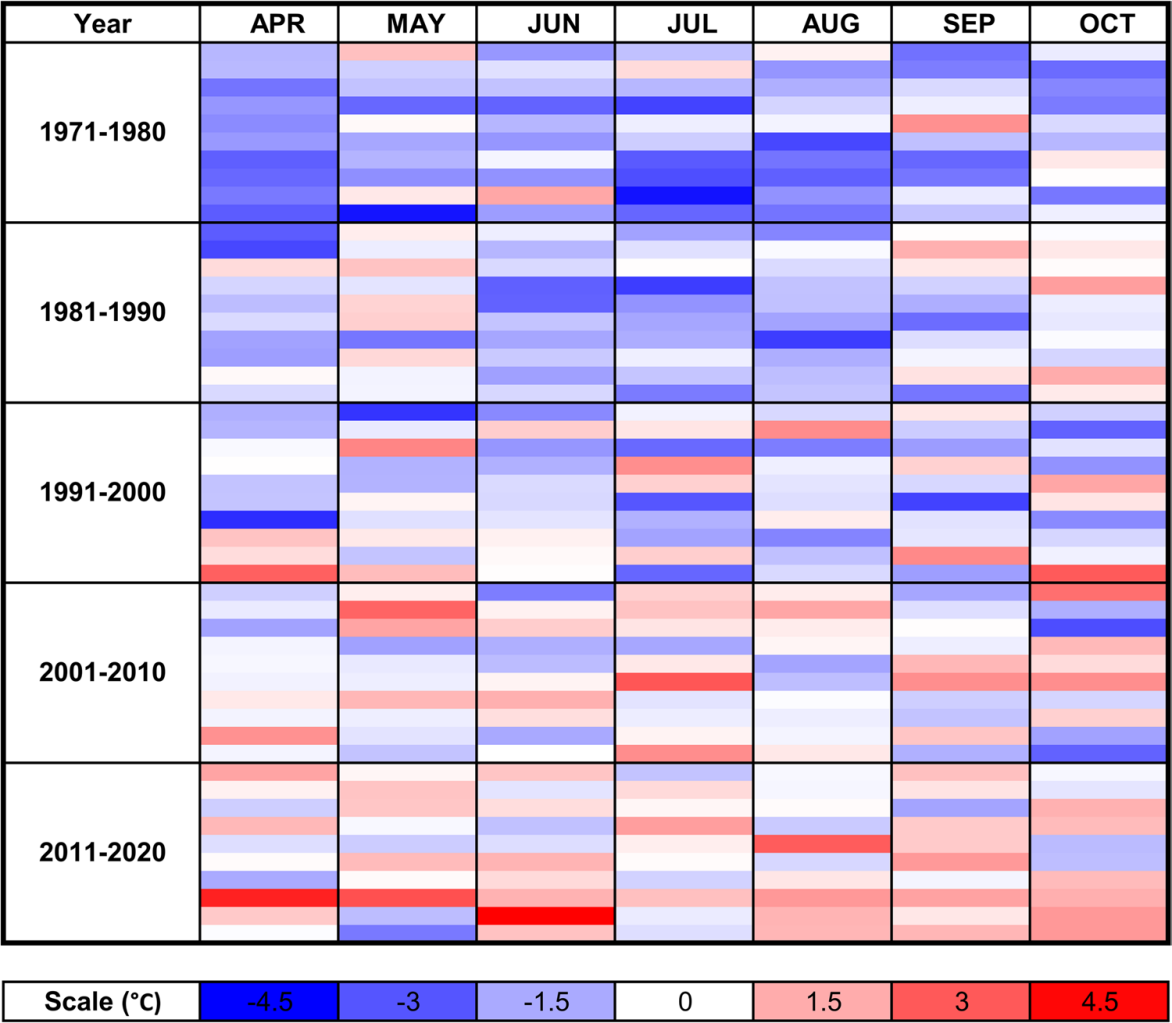
Mean grid-based weighted month temperature anomaly recorded in Poland in the period 1971–2020. Data source: Polish Institute of Meteorology and Water Management—National Research Institute, IMGW-PIB (https://dane.imgw.pl). IMGW-PIB data has been processed by Piotr Djaków model POLTEMP 1.0H8 (https://meteomodel.pl). Reference period 1991–2020, grid 4.0 km

Apart from evidencing the high applicability of developed marker array for selection of germplasm with desired agronomic traits, the present study revealed high adaptive potential of soybean as a crop for Central Europe. With the aid of molecular selection, several candidate high-yielding accessions were identified and are already undergoing performance and yield testing in different Polish agricultural conditions. The observed trend of higher yield of soybean accessions expressing intermediate phenology than those with early flowering phenotype converges with the results presented by Polish Research Centre for Cultivar Testing (COBORU) from the recent 3-year field trials performed in 2020–2022. Indeed, cultivars with intermediate phenology, requiring more than 150 days from sowing to harvest maturity, out-yielded early and very early genotypes by 30–40% in every studied year (Osiecka 2022). Analogous conclusion has been raised for simulated soybean yield potential in Northern France (about 50°N) (Boulch et al. 2021). Thus, a correct alignment of selected cultivars with the length of growing season was raised as the most critical issue in making soybean yields to be economically attractive in European locations at similar latitudes as Poland (Coleman et al. 2021). Although first attempts to cultivate soybean in Central Europe were made in the nineteenth century (Haberlandt 1878), soybean potential production is still untapped by local farmers (Karges et al. 2022). Soybean is currently (in 2022) cultivated on nearly 48,000 hectares (ha) in Poland (https://rejestrupraw.arimr.gov.pl/#), being overtopped in legume family even by peas and lupins, harvested yearly on about 50,000 and 250,000 ha, respectively (Bojarszczuk and Księżak 2020). Short growing season combined with a long-day photoperiod (about 16 h) were the main factors hampering soybean cultivation in the region during the first attempts undertaken a hundred years ago. The response of short-day plants, such as soybean, to day length increases with the increasing latitude; flowering is delayed and vegetation period is longer, leading to problems with maturation and harvesting before the end of the growing season (Garner 1933; Scott and Aldrich 1983). However, in recent 70 years, Central Europe has experienced rapid increase of air and soil surface temperature, reaching currently in spring months March, April, and May about 2–2.5 °C above the 1950s level (Kempf 2023). In Poland, mean grid-based weighted temperature in April–June in the last decade was 2.03 °C higher than in 1970s, revealing a relatively stable increase of about 0.50 ± 0.13 °C per decade (Fig. 7). Increase of air temperature resulted in an extension of the growing season in Poland by an average of 2.5 days per decade during 1951–2010, reaching currently more than 230 days that include a period of about 160–170 continuous frost-free days (Wypych et al. 2017).

As global warming is currently accelerating (Jenkins et al. 2022), thermal conditions in Central Europe are expected to be more favorable for soybean cultivation, and the length of growing season should not be a limiting factor for this crop in the near future. In 1970s, just before observed acceleration of global climate change, it was considered that soybean varieties developed for the region of Poland should be characterized by a relatively short vegetation period (125–135 days) (Szyrmer and Federowska 1975). Currently, in Polish testing trials, the highest yields are observed in soybean cultivars requiring more than 150 days of growing season, which roughly corresponds to the length of frost-free season at the test sites (Koźmiński et al. 2023; Osiecka 2022). Thus, it can be concluded that to maximize the yield, required length of growing season for soybean cultivar should at a given site align with the length of the frost-free period, which falls in Poland within the range of ~ 155 to ~ 195 days with an increasing trend of 3–6 days per decade (Koźmiński et al. 2023). Projections suggest a substantial increase in potential soybean productivity in Central Europe, with significant share of the adaptation effect in the total yield gain (exceeding 50%) by the mid-century resulting from cultivation of a long-maturing variety (Nendel et al. 2023). In our study, the highest mean yields were observed in the third year (2020), characterized by the lowest number of growing degree days (GDD) in the first 50 days from sowing (Table 5). Soybean responded to lower temperatures by significant extension of vegetative phase, reaching similar or even higher GDD value at flowering time than in warmer 2018 and 2019 seasons. It should be also noted that the 2020 growing season was characterized by higher precipitation than the two previous years, especially in the period May–September (570.1 mm vs 308.0 mm in 2018 and 259.5 mm in 2019); see Supplementary Table S2. Significant correlations between precipitation and yield related traits were confirmed in our study (Fig. 1, Supplementary Table S5); nevertheless, differences in precipitation do not explain observed significant differences in yield-related traits between 2018 and 2019.

**Table 5:** Growing degree days (GDD) calculated for field trials (base temperature 10 °C)

Year	GDD until 50 days from sowing	GDD until mean flowering date	GDD until 150 days from sowing	GDD until mean maturity date
2018	338.8	377.8	1342.5	1351.1
2019	273.2	426.7	1224.1	1238.2
2020	189.1	428.9	1103.1	1169.9

The two other traits that could significantly influence soybean yield in European climate are pod shattering and growth determination. Pod dehiscence has been widely targeted by legume breeders in Europe, including narrow-leafed lupin, yellow lupin, common bean, pea, and lentil (Parker et al. 2021; Święcicki et al. 2000). It should be noted that in extreme conditions of drought and high temperature, as observed in Poland in 1994, even lupin cultivars carrying two major non-shattering genes showed some degree of pod dehiscence (Święcicki and Święcicki 1995). Thus, July 1994 was just the second equal warmest Julies in Poland during the 240-year observation series (tying with 1834); however, both records were already surpassed three times in the first two decades of the twenty-first century according to the mean grid-based weighted month temperature anomaly POLTEMP 1.0H8 data (https://meteomodel.pl). Indeed, Europe has been recently identified as a heatwave hotspot, exhibiting three-to-four times faster heatwave trends compared to the rest of the northern mid-latitudes during the last four decades (Rousi et al. 2022). Recent projection of climate change impacts on temperature and precipitation in Central Poland revealed that temperature will be higher in all seasons as compared to the reference period (1990–2014), whereas total precipitation will rise in autumn (September–November) and winter (December–February), decrease in summer (June–August), and present unclear trend for spring (Ghazi et al. 2023). As we already observe significant correlations between soybean yield and precipitation in summer in current climate, realization of this projection may result in unsatisfactory future yields despite reaching full adaptation to the length of growing season.

Hot and dry periods in the future are likely to occur more frequently (Ault 2020; Nendel et al. 2023). Drought in summer and early autumn (i.e., at the stage of maturity) may facilitate pod dehiscence, whereas determinate growth habit may result in development of pods below the height threshold for harvesting machinery, especially in dry seasons as drought stress reduces soybean plant height at all (Mak et al. 2014). Therefore, soybean cultivars introduced into European agronomy should carry non-dehiscence alleles of both pod-shattering genes *SHAT1-5* and *qPHD1*, accompanied by an indeterminate growth habit allele of *Dt1*.

## Conclusions

Our results provide strong evidence on high adaptive potential of soybean as a crop for Central Europe that could be exploited with the aid of molecular selection. Thus, we demonstrated high applicability of PCR array for marker-assisted breeding of soybean towards adaptation to Polish agroclimatic conditions.

Moreover, the current research illustrated the strong effect of photoperiod on soybean performance, highlighting also significant associations for growth habit and pod dehiscence. To achieve the highest yields, breeders should focus on indeterminate and non-shattering accessions with the required length of growing season almost equally matching the length of frost-free season. Briefly, it requires selection towards dominant *Dt1*, *E3*, and *E4* alleles and the recessive *qPHD1* allele.

## Supplementary Information

Below is the link to the electronic supplementary material.

Supplementary file1 (PDF 939 KB)

Supplementary file2 (XLSX 151 KB)

## Data Availability

All data generated or analyzed during this study are included in this published article and its supplementary information files.

## Declarations

### Disclaimer

The funder had no role in the design of the study; in the collection, analyses, or interpretation of data; in the writing of the manuscript, or in the decision to publish the results.
