# Supplementary material for: Molecular selection of soybean towards adaptation to Central European agroclimatic conditions
Source: J Appl Genet. 2024 Jul 2;66(1):29–45. doi: 10.1007/s13353-024-00889-6 (PMC11761526; doi:10.1007/s13353-024-00889-6)

Sandra Rychel-Bielska, Michał Książkiewicz, Danuta Kurasiak-Popowska, Agnieszka Tomkowiak, Wojciech Bielski, Dorota Weigt, Janetta Niemann, Anna Surma, Bartosz Kozak, Jerzy Nawracała

Molecular selection of soybean towards adaptation to Central European agroclimatic conditions

Journal of Applied Genetics

Supplementary Figure 1. Agarose gel electrophoregrams showing polymorphism of PCR-based markers targeting early maturity, pod shattering and growth determination genes.

Dt1_1_SNP_CAPS_*NdeI*

A – Dt1 and other: 193 + 217 bp

B – dt1-b: 410 bp

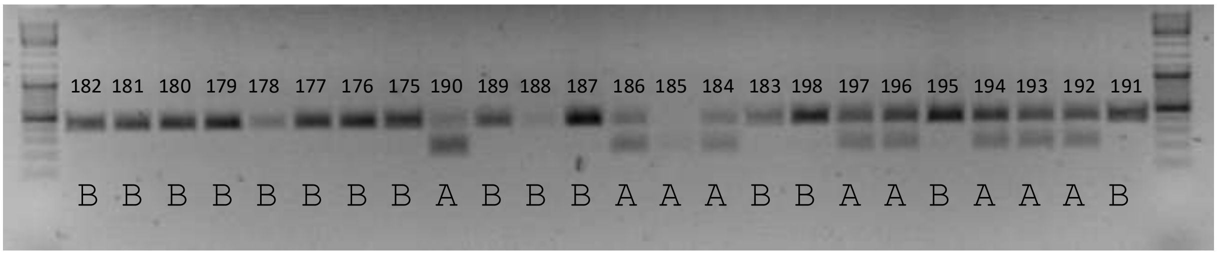

Dt1_2_INDEL_PCR

A – Dt1 and other: 325 bp

B – dt1-b: 319 bp

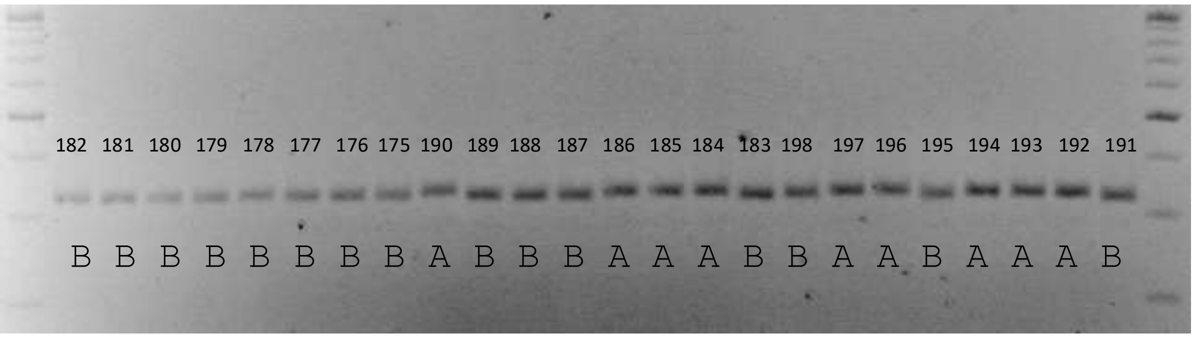

Dt1_3_SNP_CAPS_ *AflI*

A – Dt1 and other: 201 + 123 bp

B – dt1-b: 319 bp

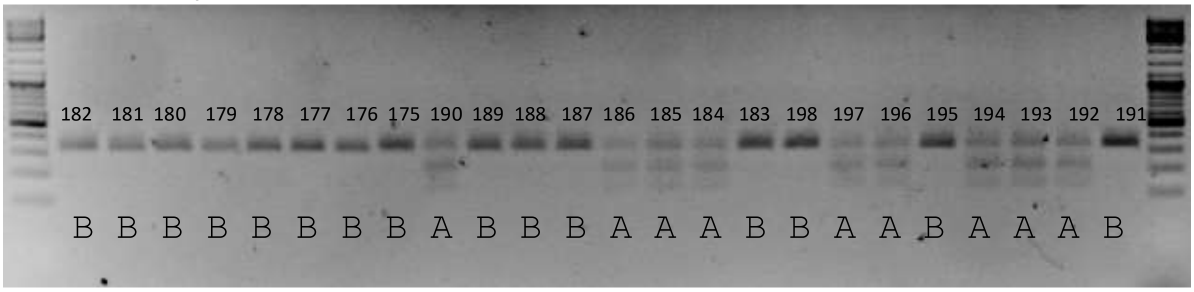

Dt1_4_SNP_dCAPS_*XbaI*

A – Dt1 and other: 155 + 21 bp

B – dt1-ab: 176 bp

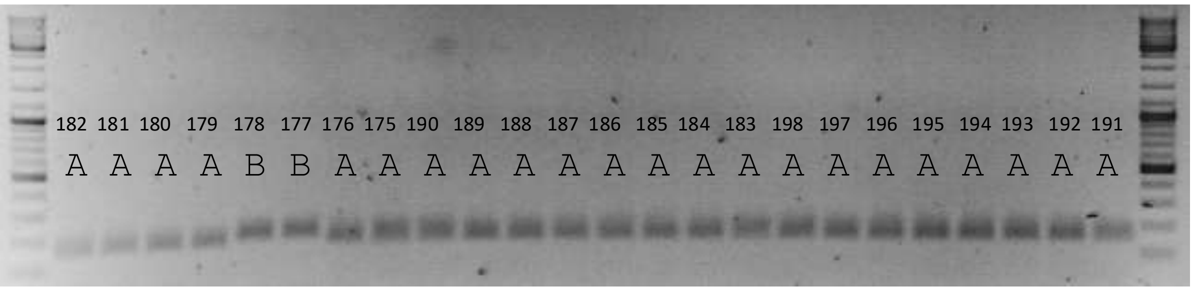

Dt1_5_SNP_CAPS_*HindIII*

A – Dt1 and other: 808 bp

B – dt1-bb: 215 + 593bp

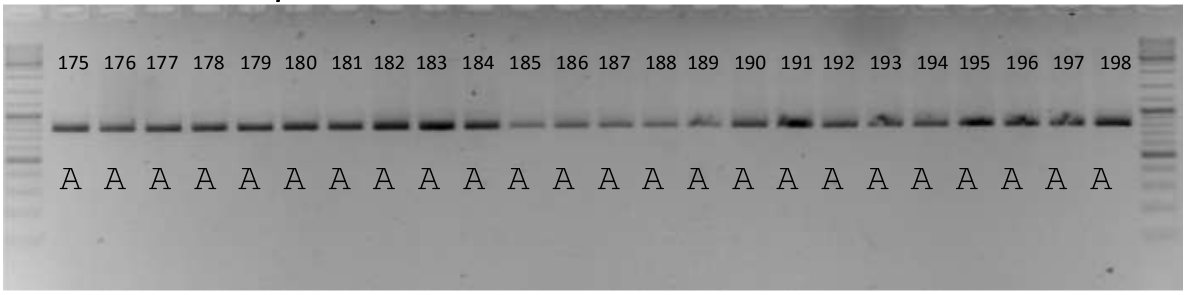

Dt1_6_SNP_CAPS_*AccI*

A – Dt1 and other: 646 + 92 + 70 bp

B – dt1-tb: 738 + 70 bp

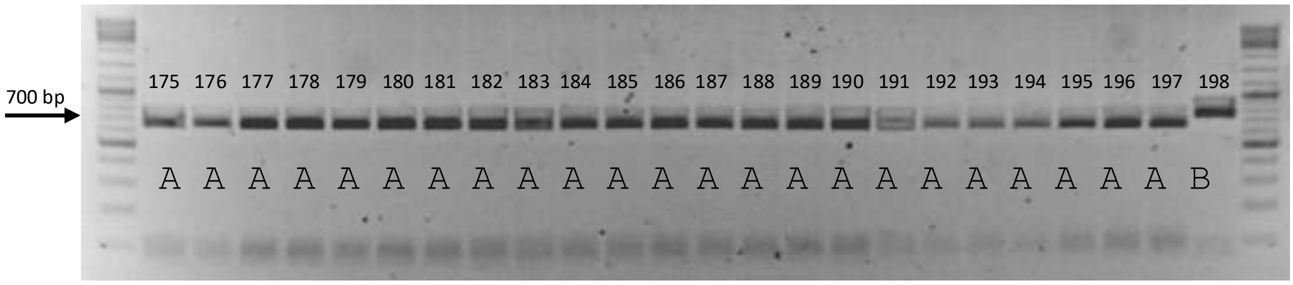

Dt1_36_SNP_CAPS_*AvaII*

A – Dt1 and other: 229 + 97 bp

B – dt1-ta: 326 bp (present only in a control line)

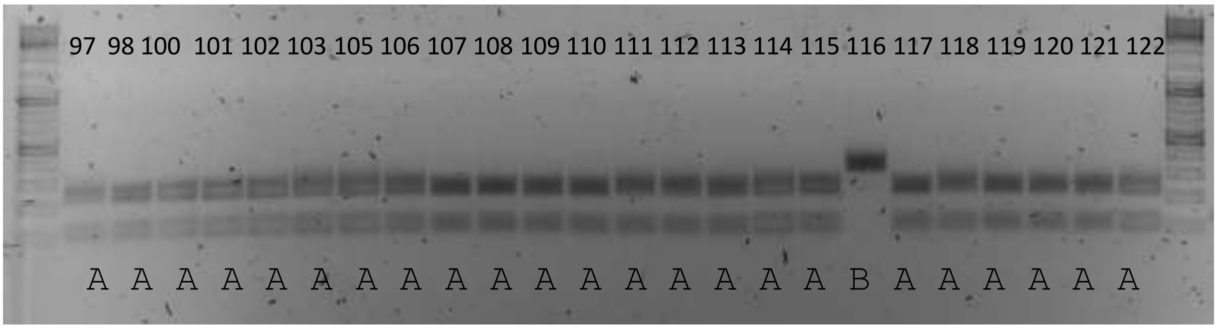

Dt2_7_SSR_PCR

A – Dt2: other products

B – dt2: 280 bp

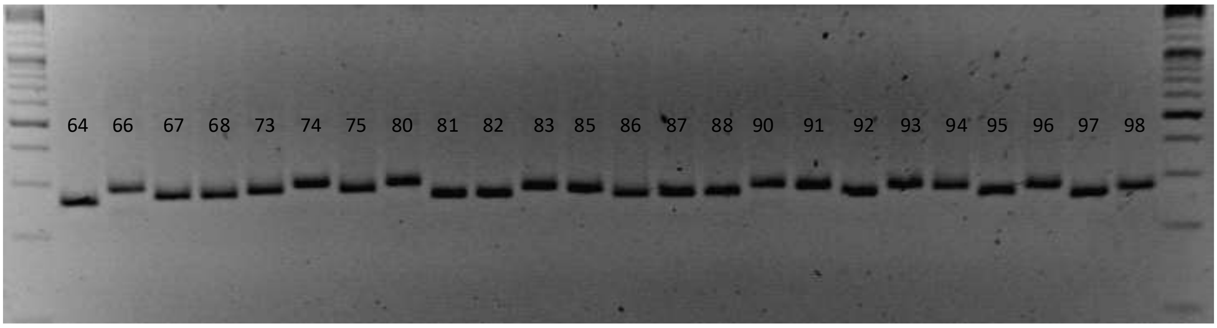

Dt2_8_SSR_PCR

A – Dt2: other products

B – dt2: 221 bp

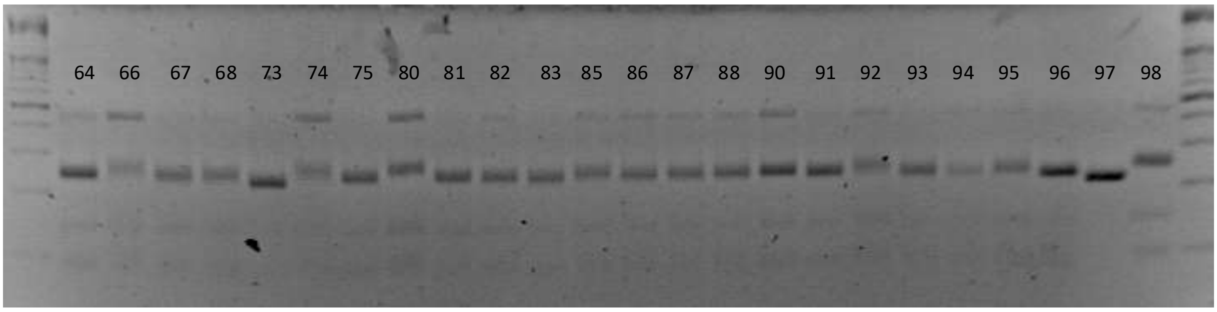

Dt2_9_SSR_PCR

A – Dt2: other products

B – dt2: 202 bp

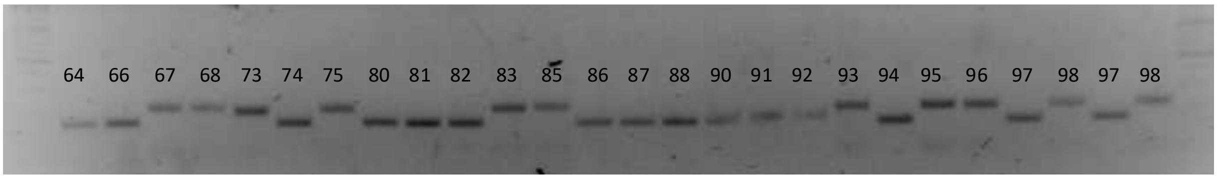

E1_12_SNP_dCAPS_*TaqI*

A – E1/e1-fs/e1-nl: 443 or 444 bp

B – e1-as: 413 + 31 bp

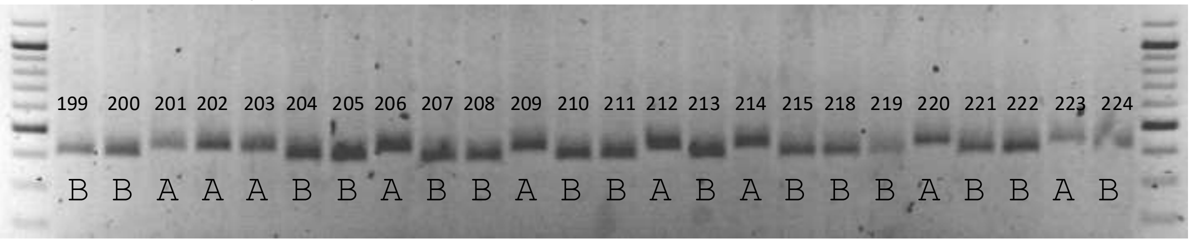

E1_13_SNP_CAPS_*HinfI*

A – E1/e1-as: 186 + 36 bp

B – e1-fs: 146 + 46 + 36 bp

C – e1-nl: faint products

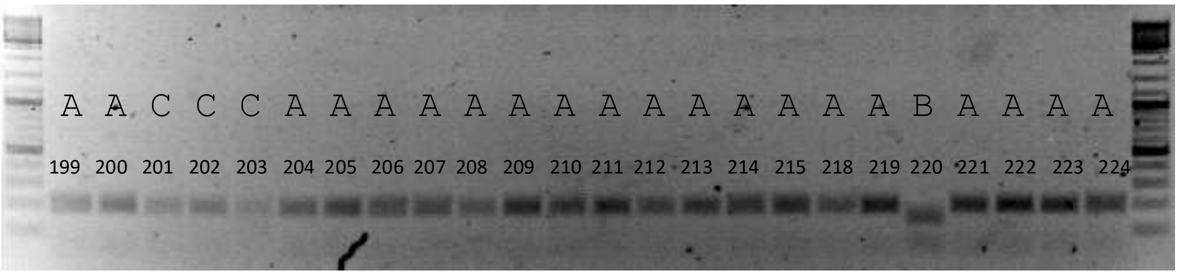

E1_37_INDEL_PCR

A – E1/e1-as/e1-fs: 841 or 840 bp

B – e1-re: 592 bp

C – e1-nl: no product

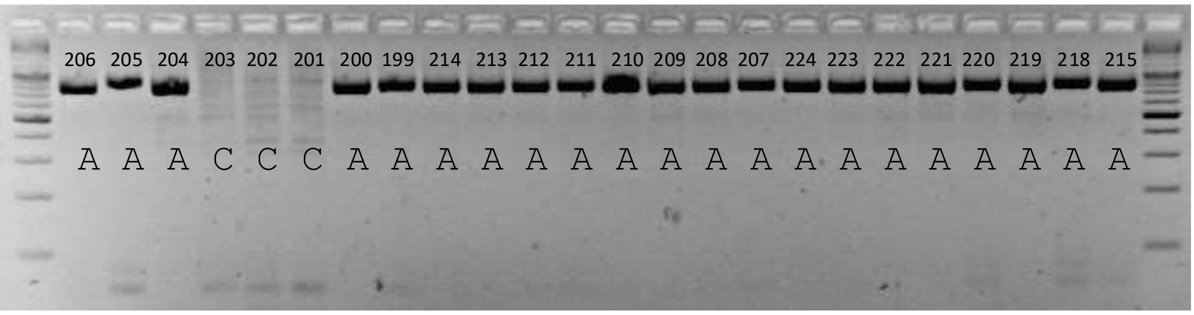

E2_15_SNP_dCAPS_*DraI*

A – E2: 130 bp

B – e2: 27 + 103 bp

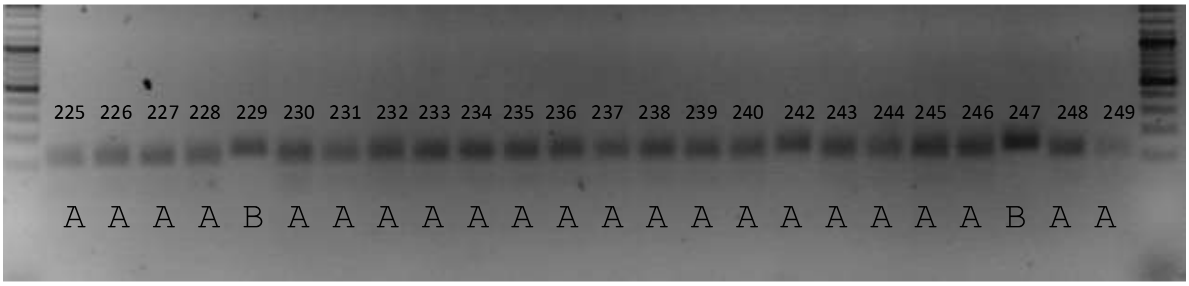

E2_17_INDEL_PCR

A – E2-in: 548 bp

C – E2-dl: 512 bp

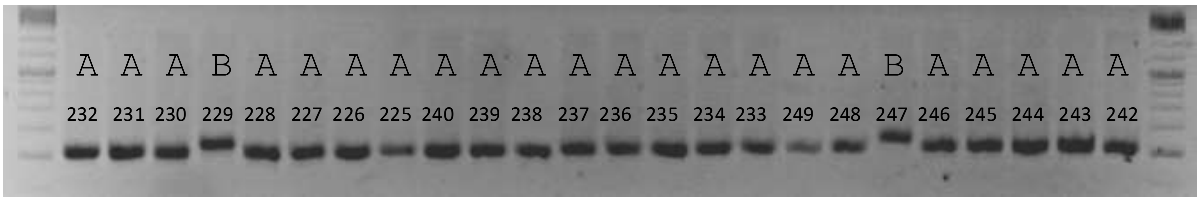

E3_18_INDEL_PCR

C – E3-Misuzudaizu: 1339 bp

A – E3-Harosoy/Moshidougong: 558 bp

B – e3-tr: 275 bp

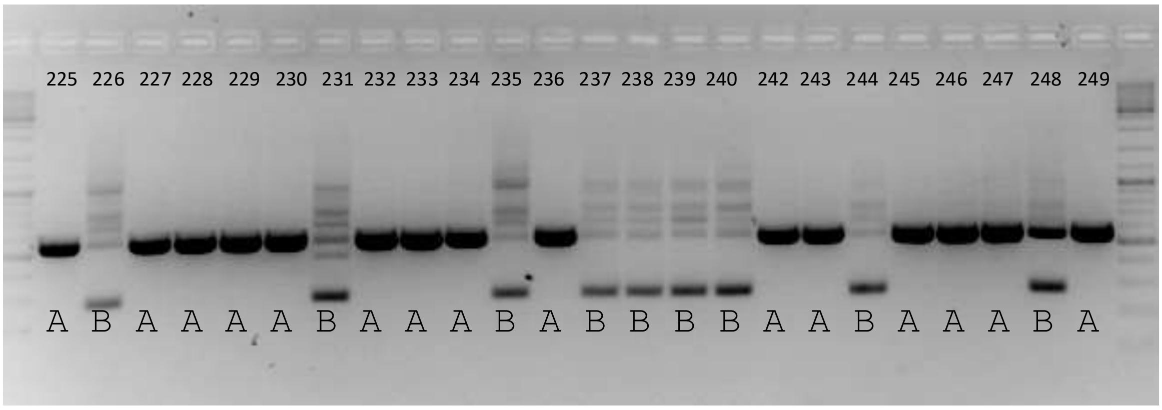

E3_19_SNP_CAPS_*MseI*

A – E3-Harosoy and other: 324 bp

B – E3-Moshidougong: 223 + 101 bp

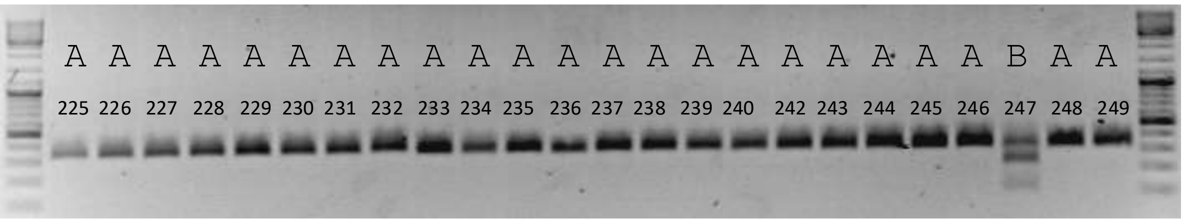

E3_20_SNP_CAPS_*AleI*

A – E3 and other: 552 + 206 bp

B – e3-fs: 759 bp

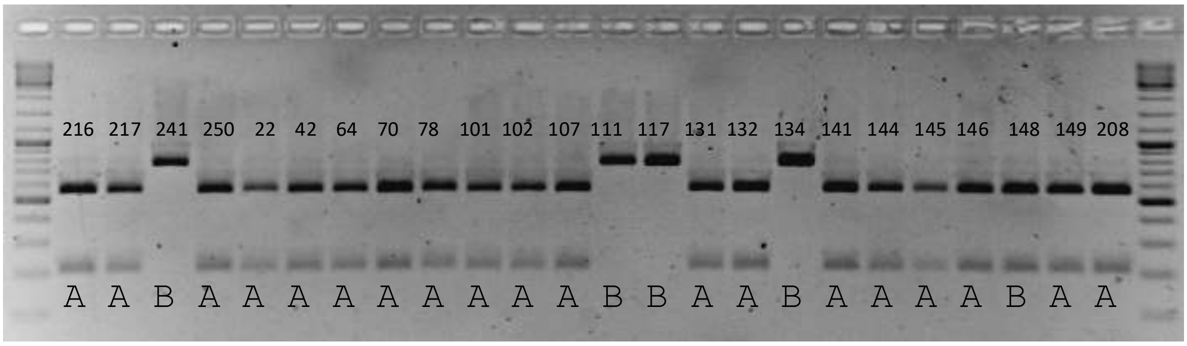

E3_21_SNP_dCAPS_*MfeI*

A – E3 and other: 163 bp

B – e3-ns: 140 + 23 bp

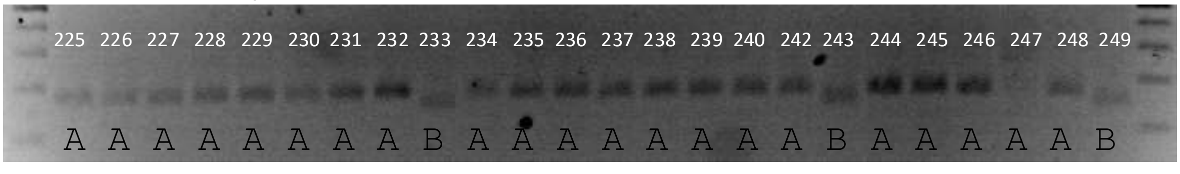

E4_22_INDEL_PCR

A – E4 and other: 1229 bp

B – e4-SORE-1: 837 bp

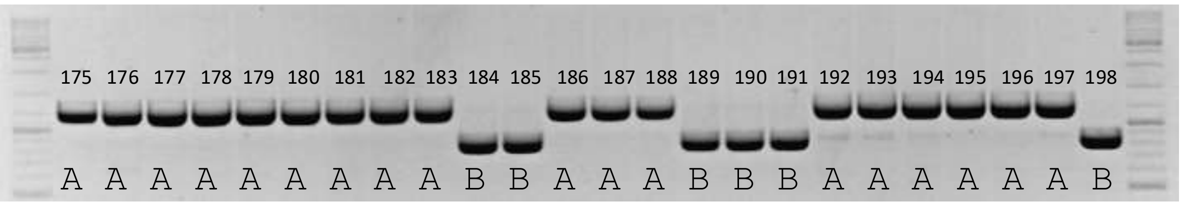

E4_23_SNP_CAPS_*AlfII*

A – E4 and other: 494 bp

B – e4-kam: 286 + 208 bp (present only in a control line)

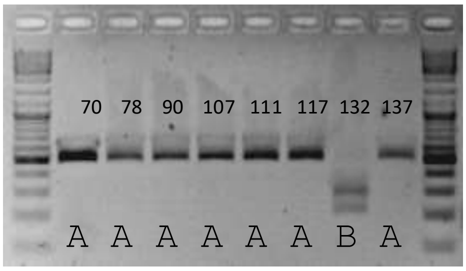

E4_24_SBP_CAPS_*BspHI*

A – E4 and other: 494 bp

B – e4-kes: 399 + 95 bp

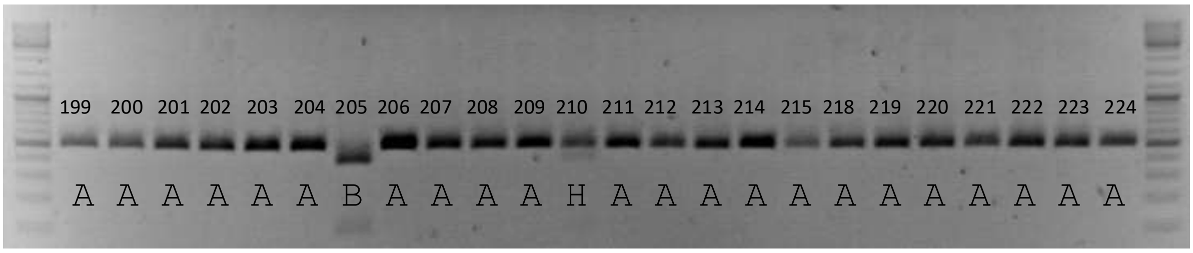

E4_25_CAPS_*SacI*

A – E4 and other: 535 bp

B – e4-oto: 439 + 96 bp

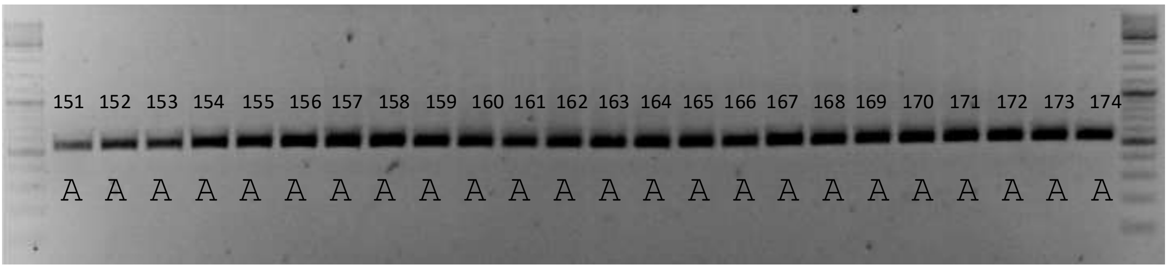

E4_26_dCAPS_*EcoRV*

A – E4 and other: 355 bp

B – e4-tsu: 23 + 332 bp

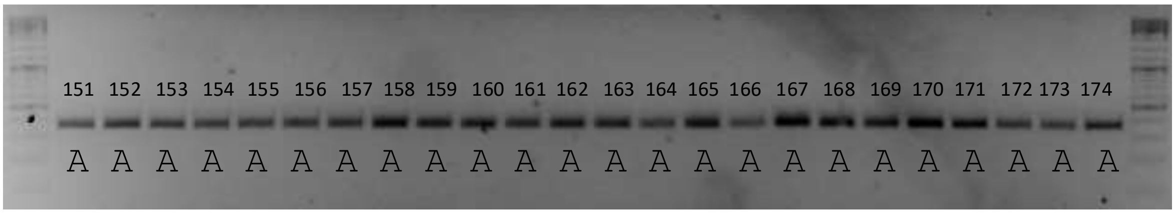

E7_27_SSR_PCR

A – E7: 168 bp

B – e7: other products

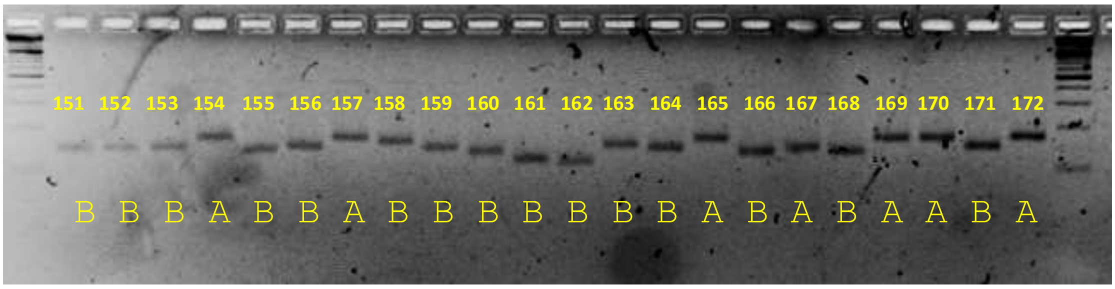

E9_28_INDEL_PCR

A – E9Harosoy: 187 bp

B – other: 230 bp

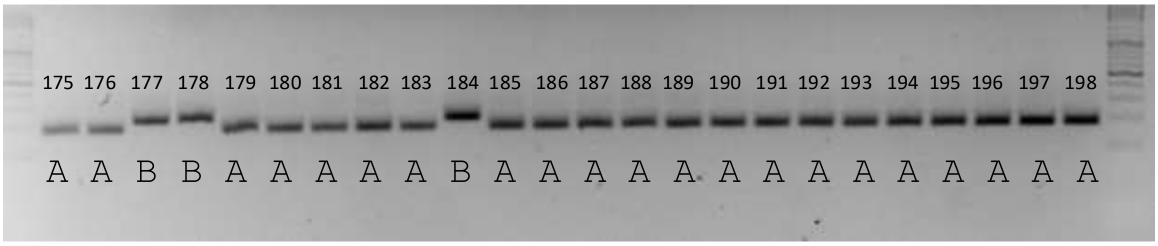

E9_29_INDEL_PCR

A – other: 144 bp

B – E9indel10: 134 bp

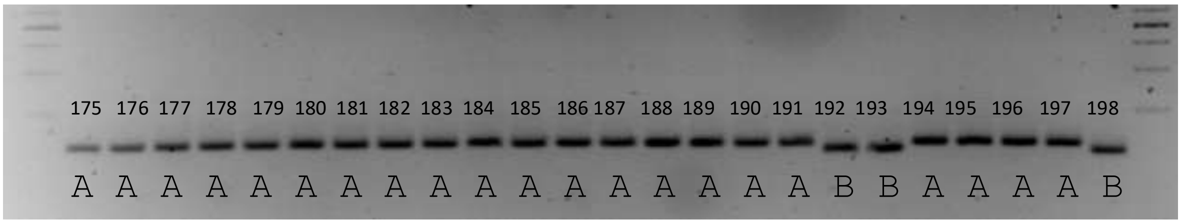

E9_30_SNP_dCAPS_*HinfI*

A – other: 160 bp

B – E9SNP#17: 138 bp

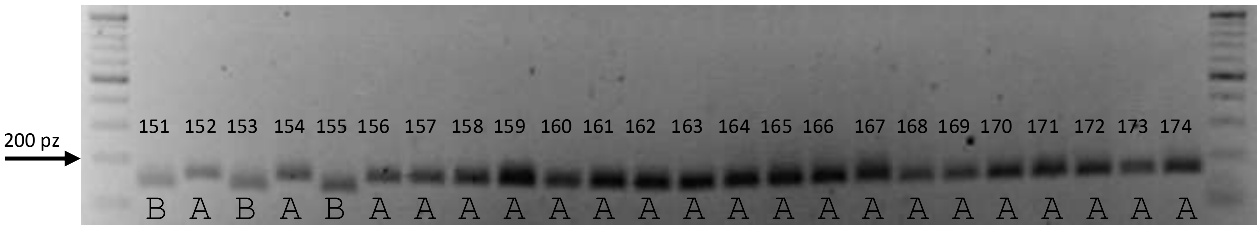

E9_31_SORE-1_INDEL_PCR

A – other: 440 bp

B – e9_SORE-1: 306 bp

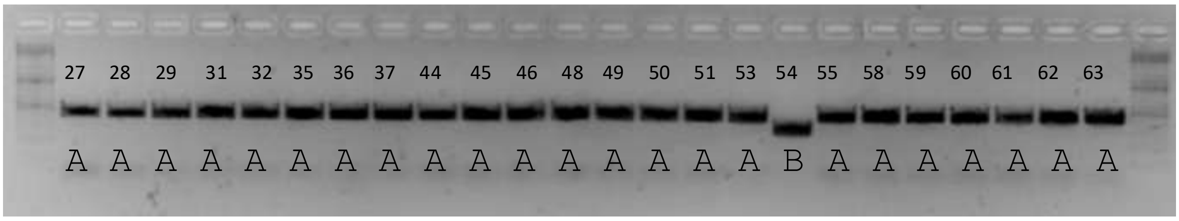

E10_34_SNP_CAPS_*BfaI*

A – E10: 78 i 38 bp

B – e10_exonSNP: 116 bp

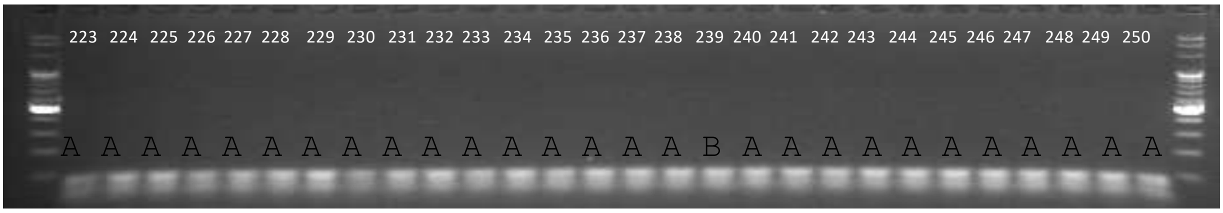

E10_35_SNP_dCAPS_*TaqI*

A – E10: 110 bp

B – e10_3'UTR_SNP: 82 +28 bp

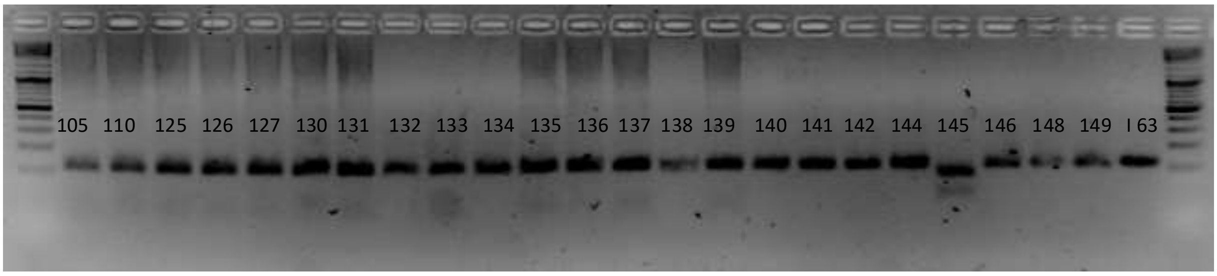

qPHD1_38_SNP_dCAPS_*StyI*

A – KSS-SNP5(A): 75 bp (non-shattering)

B – KSS-SNP5(G): 24 + 51 bp (shattering)

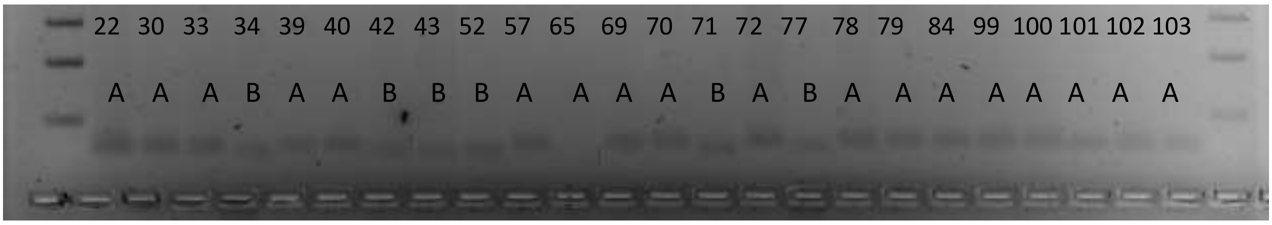

Supplement: Supplementary file 1 — Supplementary file1 (PDF 939 KB) [file 13353_2024_889_MOESM1_ESM.pdf]
